# Signalling, trafficking and glucoregulatory properties of glucagon‐like peptide‐1 receptor agonists exendin‐4 and lixisenatide

**DOI:** 10.1111/bph.15134

**Published:** 2020-06-19

**Authors:** Philip Pickford, Maria Lucey, Zijian Fang, Stavroula Bitsi, Jorge Bernardino de la Serna, Johannes Broichhagen, David J. Hodson, James Minnion, Guy A. Rutter, Stephen R. Bloom, Alejandra Tomas, Ben Jones

**Affiliations:** ^1^ Section of Endocrinology and Investigative Medicine Imperial College London London UK; ^2^ Section of Cell Biology and Functional Genomics Imperial College London London UK; ^3^ National Heart and Lung Institute Imperial College London London UK; ^4^ Department Chemical Biology Max Planck Institute for Medical Research Heidelberg Germany; ^5^ Department Chemical Biology Leibniz‐Forschungsinstitut für Molekulare Pharmakologie (FMP) Berlin Germany; ^6^ Institute of Metabolism and Systems Research (IMSR), and Centre of Membrane Proteins and Receptors (COMPARE) University of Birmingham Birmingham UK; ^7^ Centre for Endocrinology, Diabetes and Metabolism Birmingham Health Partners Birmingham UK

## Abstract

**Background and Purpose:**

Amino acid substitutions at the N‐termini of glucagon‐like peptide‐1 (GLP‐1) receptor agonist peptides result in distinct patterns of intracellular signalling, sub‐cellular trafficking and efficacy in vivo. Here, we to determine whether sequence differences at the ligand C‐termini of clinically approved GLP‐1 receptor agonists exendin‐4 and lixisenatide lead to similar phenomena.

**Experimental Approach:**

Exendin‐4, lixisenatide and N‐terminally substituted analogues with biased signalling characteristics were compared across a range of in vitro trafficking and signalling assays in different cell types. Fluorescent ligands and new time‐resolved FRET approaches were developed to study agonist behaviours at the cellular and sub‐cellular level. Anti‐hyperglycaemic and anorectic effects of each parent ligand and their biased derivatives were assessed in mice.

**Key Results:**

Lixisenatide and exendin‐4 showed equal binding affinity, but lixisenatide was fivefold less potent for cAMP signalling. Both peptides induced extensive GLP‐1 receptor clustering in the plasma membrane and were rapidly endocytosed, but the GLP‐1 receptor recycled more slowly to the cell surface after lixisenatide treatment. These combined deficits resulted in reduced maximal sustained insulin secretion and reduced anti‐hyperglycaemic and anorectic effects in mice with lixisenatide. N‐terminal substitution of His1 by Phe1 to both ligands had favourable effects on their pharmacology, resulting in improved insulin release and lowering of blood glucose.

**Conclusion and Implications:**

Changes to the C‐terminus of exendin‐4 affect signalling potency and GLP‐1 receptor trafficking via mechanisms unrelated to GLP‐1 receptor occupancy. These differences were associated with changes in their ability to control blood glucose and therefore may be therapeutically relevant.

**Abbreviations**
βarr2: β‐arrestin‐2
DERET: diffusion‐enhanced resonance energy transfer
EGFR: EGF receptor
Ex4: exendin‐4
FITC: fluorescein isothiocyanate
HTRF: homogenous time‐resolved fluorescence
Lixi: lixisenatide
RICS: raster image correlation spectroscopy
TMR: tetramethylrhodamine
TR‐FRET: time‐resolved FRET
Veh: vehicle

**What is already known**

- Glucagon‐like peptide‐1 receptor agonists are used to treat type 2 diabetes and obesity.
- Recently described biased GLP‐1 receptor agonists show distinct patterns of intracellular signalling and membrane trafficking.

**What this study adds**

- Two commonly prescribed GLP‐1 agonists, exendin‐4 and lixisenatide, perform differently in vitro and in vivo.
- These differences may be linked to their distinct effects on GLP‐1 receptor recycling.

**What is the clinical significance**

- Signal bias and trafficking should be considered in the development of novel GLP‐1 agonists.

## 1 INTRODUCTION

The glucagon‐like peptide‐1 (GLP‐1) receptor is a well‐established pharmacological target for the treatment of both type 2 diabetes and obesity due to its beneficial effects on weight loss and pancreatic beta cell function (Andersen, Lund, Knop, & Vilsbøll, 2018). The main endogenous ligand for GLP‐1 receptor, the 29 amino acid peptide GLP‐1(7‐36)NH_2_, is highly susceptible to degradation by proteolytic enzymes that rapidly destroy it in the circulation, making it unsuitable as a therapeutic agent (Deacon et al., 1998). Therefore, a number of synthetic GLP‐1 agonists with longer circulatory half‐lives have been developed and subsequently approved for human use (de Graaf et al., 2016). One example is the GLP‐1 homologue peptide exendin‐4 (Eng, Kleinman, Singh, Singh, & Raufman, 1992), in clinical use for type 2 diabetes treatment as exenatide. This molecule features an extended, proline‐rich C‐terminal extension (sequence GAPPPS‐NH_2_), which is absent in GLP‐1 itself. The precise role of this feature is not clear, but various possibilities have been suggested, including stabilisation of the peptide helical structure (Neidigh, Fesinmeyer, Prickett, & Andersen, 2001), facilitation of inter‐protomer coupling within receptor oligomers (Koole et al., 2017) and protection against enzymatic degradation (Lee et al., 2018). A further approved type 2 diabetes GLP‐1 mimetic peptide, lixisenatide, shares the first 37 amino acids with exendin‐4, including most of the GAPPPS sequence but includes an additional six lysine residues at the C‐terminus prior to the terminal amidation (Andersen et al., 2018). Due to putative importance of the exendin‐4 C‐terminus, it is conceivable that the lixisenatide‐specific changes could affect its pharmacology.

Biased signalling has emerged as a promising strategy to improve the therapeutic efficacy of drugs through selective activation of “beneficial” intracellular pathways, while minimising those thought to be responsible for adverse effects (Kenakin, 2018). Recent work has highlighted how GLP‐1 receptor signal bias and related membrane trafficking effects regulate insulin release from beta cells (Zhang et al., 2015; Buenaventura et al., 2018; Jones, Buenaventura, et al., 2018). Following agonist binding the GLP‐1 receptor is rapidly endocytosed and while active GPCRs can continue to generate intracellular signals within the endosomal compartments (Eichel & von Zastrow, 2018), the availability of surface GLP‐1 receptors to extracellular ligand appears to be an important determinant of sustained insulinotropic efficacy in a pharmacological setting (Jones, Buenaventura, et al., 2018). The GLP‐1 receptor ligand N‐terminus interacts with the receptor core to instigate conformational rearrangements needed for stable engagement with intracellular signalling and trafficking effectors, while its C‐terminus facilitates this process by establishing the correct orientation of the peptide through interactions with the receptor extracellular domain (de Graaf et al., 2016). Suggesting that the C‐terminal sequence differences between exendin‐4 and lixisenatide might impact on these cellular processes, a limited evaluation in our earlier study implied that lixisenatide displays reduced signalling potency and insulinotropism compared to exendin‐4 (Jones, Buenaventura, et al., 2018).

In the present study, we extended our earlier evaluation to include formal comparison of bias between cAMP signalling and endocytosis in different cell types, measurements of ligand‐induced clustering at the plasma membrane, post‐endocytic targeting to recycling and degradative pathways aided by a novel cleavable time‐resolved FRET probe and assessment of the impact of these changes on exendin‐4 versus lixisenatide metabolic responses in vivo.

## 2 METHODS

### 2.1 Materials

All peptides and fluorescent peptide conjugates were obtained from WuXi AppTec (Wuhan) Co. Ltd. SNAP‐Lumi4‐Tb, BG‐SS‐Lumi4‐Tb and homogenous time‐resolved fluorescence (HTRF) reagents for cAMP (cAMP Dynamic 2) and insulin (Insulin High Range Assay) measurement were obtained from Cisbio. SNAP‐Surface probes were obtained from New England Biolabs. NanoGlo live cell reagents were obtained from Promega. Exendin‐4 fluorescent EIA kits were obtained from Phoenix Pharmaceuticals. All other standard laboratory chemicals or culture reagents were obtained from Sigma or Thermo Fisher unless otherwise specified. The sources of all plasmids are indicated in the relevant sections below.

### 2.2 Cell culture

HEK293 cells (RRID:CVCL_0045) stably expressing human SNAP‐GLP‐1 receptor (“HEK293‐SNAP‐GLP‐1 receptor”) were generated by transfection of a SNAP‐GLP‐1 receptor vector (Cisbio) followed by G418 selection and maintained in DMEM with 10% FBS, 1% penicillin/streptomycin and G418 (1 mg·ml^−1^). HEK293T (RRID:CVCL_0063) cells were maintained similarly but without G418. Monoclonal CHO‐K1 cells stably expressing human SNAP‐GLP‐1 receptor (“CHO‐K1‐SNAP‐GLP‐1 receptor”) were generated as above, with single clones obtained by FACS and subsequently expanded; cells were maintained in DMEM with 10% FBS, 1% non‐essential amino acids, 20‐mM HEPES, 1% penicillin/streptomycin and 1 mg·ml^−1^ G418. Wild type INS‐1832/3 cells (Hohmeier et al., 2000) (a gift from Prof. Christopher Newgard, Duke University) were maintained in RPMI‐1640 with 11‐mM glucose, 10‐mM HEPES, 2‐mM glutamine, 1‐mM sodium pyruvate, 50‐μM β‐mercaptoethanol, 10% FBS and 1% penicillin/streptomycin. INS‐1832/3 cells lacking endogenous GLP‐1 receptor after CRISPR/Cas9 deletion (Naylor et al., 2016) (a gift from Dr Jacqueline Naylor, Astra Zeneca) were used to generate a polyclonal population expressing human SNAP‐GLP‐1 receptor by G418 selection and were maintained as for wild type INS‐1832/3 cells, with the addition of G418 (1 mg·ml^−1^). MIN6B1 cells (Lilla et al., 2003) (a gift from Prof Philippe Halban, University of Geneva) were maintained in DMEM with 15% FBS, 50‐μM β‐mercaptoethanol and 1% penicillin/streptomycin. Transfections were performed using Lipofectamine 2000 according to the manufacturer's instructions.

### 2.3 Time resolved (TR)‐FRET surface receptor binding assays

HEK293‐SNAP‐GLP‐1 receptor cells were labelled in suspension with SNAP‐Lumi4‐Tb (40 nM) for 1 h at room temperature in complete medium. After washing and resuspension in HBSS containing 0.1% BSA and metabolic inhibitors (20 mmol·L^−1^ 2‐deoxygucose and 10 mmol·L^−1^ NaN_3_) to prevent GLP‐1 receptor internalisation (Widmann, Dolci, & Thorens, 1995), binding experiments were performed using fluorescein isothiocyanate (FITC)‐conjugated ligands as described below, with TR‐FRET measured in a Flexstation 3 plate reader (Molecular Devices) using the following settings: λ_ex_ = 335 nm, λ_em_ = 520 and 620 nm, delay 50 μs, integration time 400 μs. Binding was quantified as the ratio of fluorescent signal at 520 nm to that at 620 nm, after subtraction of ratio obtained in the absence of FITC‐ligands.

#### 2.3.1 Saturation binding experiments with fluorescein isothiocyanate (FITC)‐ligands

Cells were treated with FITC‐ligands over a range of concentrations for 24 h at 4°C before measurement. Equilibrium binding constants were calculated using the “one site‐specific binding” algorithm in Prism 8 (GraphPad Software).

#### 2.3.2 Competition binding experiments at equilibrium

Cells were treated with a fixed concentration (10 nM) of exendin(9–39)‐FITC in competition with a range of concentrations of unlabelled exendin‐4 or lixisenatide for 24 h at 4°C before measurement. Binding constants were calculated using the “one site‐fit K_i_” algorithm in Prism 8, using the equilibrium dissociation constant for exendin(9–39)‐FITC measured in the same experiment by saturation binding.

#### 2.3.3 Competition kinetic binding experiments

TR‐FRET signals were measured at regular intervals before and after addition of different concentrations of exendin(9–39)‐FITC, or different concentrations of unlabelled agonist in combination with a fixed concentration (10 nM) of exendin(9–39)‐FITC at 37°C. Rate constants for association and dissociation of the unlabelled ligands were calculated using the “kinetics of competition binding” algorithm in Prism 8, using values for exendin(9–39)‐FITC determined in the same experiment.

### 2.4 cAMP assays by homogenous time‐resolved fluorescence (HTRF)

Cells were stimulated with agonist for the indicated period in their respective growth mediums without FBS. Assays were performed at 37°C without PDE inhibitors, except for with INS‐1832/3 and MIN6B1 cells, where IBMX was added at 500 μM. At the end of the incubation period, cells were lysed and cAMP was determined by HTRF (cAMP Dynamic 2 kit, Cisbio) using a Spectramax i3x plate reader (Molecular Devices).

### 2.5 Dynamic signalling measurements via FRET biosensors for cAMP and PKA

HEK293T cells were transiently transfected with the FRET‐based cAMP sensor ^T^Epac^VV^ (a gift from Prof Kees Jalink, Netherlands Cancer Institute) (Klarenbeek, Goedhart, Hink, Gadella, & Jalink, 2011) using Lipofectamine 2000. CHO‐K1‐SNAP‐GLP‐1 receptor cells stably expressing the AKAR4‐NES (Herbst, Allen, & Zhang, 2011) biosensor, a gift from Prof Jin Zhang (Addgene plasmid 64,727), were used to measure cytoplasmic protein kinase A (PKA) activation. Cells were suspended in HBSS, placed into black, clear‐bottom plates and FRET was measured before and after agonist addition in a Flexstation 3 plate reader at 37°C using the following settings: λ_ex_ = 440 nm, λ_em_ = 485 and 535 nm. FRET was quantified as the ratio of fluorescent signal at 535 nm to that at 485 nm after subtraction of background signal at each wavelength. Dynamic FRET changes were expressed relative to individual well baseline to improve precision.

### 2.6 Measurement of mini‐G and β‐arrestin recruitment by NanoBiT complementation

The plasmids for mini‐G_s_, ‐G_i_ and ‐G_q_, each tagged at the N‐terminus with the LgBiT tag and the SmBiT‐tagged endothelin A receptor (ETAR) plasmid (Wan et al., 2018) were a gift from Prof Nevin Lambert, Medical College of Georgia. The plasmid for β‐arrestin‐2 fused at the N‐terminus to LgBiT was obtained from Promega (plasmid no. CS1603B118); this configuration was used due to previous success with another class B GPCR (Shintani et al., 2018). The SmBiT tag was cloned in frame at the C‐terminus of the GLP‐1 receptor by substitution of the Tango sequence on a N‐terminally FLAG‐tagged GLP‐1 receptor‐Tango expression vector (Kroeze et al., 2015), a gift from Dr Bryan Roth, University of North Carolina (Addgene plasmid # 66295). HEK293T cells in 12‐well plates were co‐transfected for 24 h with Lipofectamine 2000 using the following quantities of DNA: 0.5 μg each of GLP‐1 receptor‐SmBiT and LgBiT‐tagged mini‐G or 0.05 μg each of GLP‐1 receptor‐SmBiT and LgBiT‐tagged β‐arrestin‐2 with 0.9 μg empty vector DNA (pcDNA3.1). Cells were resuspended in Nano‐Glo dilution buffer and fumarazine (1:20) (Promega) and seeded in 96‐well half area white plates. Baseline luminescence was measured over 5 min using a Flexstation 3 plate reader at 37°C before addition of ligand or vehicle. Luminescent signal was then serially monitored over 30 min and responses were normalised to average baseline.

### 2.7 Measurement of GLP‐1 receptor internalisation by diffusion‐enhanced resonance energy transfer (DERET)

This assay was adapted from previous descriptions (Roed et al., 2014; Jones, Buenaventura, et al., 2018). HEK293‐SNAP‐GLP‐1 receptor cells were labelled in suspension with SNAP‐Lumi4‐Tb (40 nM) for 1 h at room temperature in complete medium. After washing and resuspension in 24‐μM fluorescein solution in HBSS, TR‐FRET was monitored before and after agonist addition in a Flexstation 3 plate reader at 37°C using the following settings: λ_ex_ = 335 nm, λ_em_ = 520 and 620 nm, delay 400 μs, integration time 1,500 μs. TR‐FRET was quantified as the ratio of fluorescent signal at 520 nm to that at 620 nm after subtraction of background signal at each wavelength (simultaneously recorded from wells containing 24‐μM fluorescein in HBSS but no labelled cells).

### 2.8 Measurement of GLP‐1 receptor clustering by TR‐FRET

The assay was performed similarly to a previous description (Buenaventura et al., 2019). HEK293‐SNAP‐GLP‐1 receptor cells were labelled in suspension with SNAP‐Lumi4‐Tb (40 nM) and SNAP‐Surface 649 (1 mM) for 1 h at room temperature in complete medium. After washing, cells were resuspended in HBSS, and TR‐FRET was monitored before and after agonist addition at 37°C in a Spectramax i3x plate reader in HTRF mode. TR‐FRET was quantified as the ratio of fluorescent signal at 665 nm to that at 616 nm, after subtraction of background signal at each wavelength.

### 2.9 Measurement of GLP‐1 receptor recycling by TR‐FRET

CHO‐K1‐SNAP‐GLP‐1 receptor cells adhered in white 96‐well half area tissue culture‐treated plates were labelled with BG‐SS‐Lumi4‐Tb (40 nM unless indicated otherwise) for 30 min at 37°C. BG‐SS‐Lumi4‐Tb is a cleavable SNAP‐tag probe that allows release of the lanthanide moiety following reduction of its disulfide bond when exposed to reducing agents. After washing, BG‐SS‐Lumi4‐Tb labelled cells were treated with agonist for 30 min at 37°C to induce GLP‐1 receptor internalisation, placed on ice to arrest further trafficking and residual surface GLP‐1 receptor was de‐labelled by cleavage of the lanthanide using the cell‐impermeant reducing agent 2‐mercaptoethane sulfonate (Mesna, 100 mM in TNE buffer, pH 8.6) for 5 min. After further washing, exendin(9–39)‐FITC, added as a non‐internalising FRET‐acceptor for the GLP‐1 receptor recycling to the cell surface, was added at 10 nM in HBSS and TR‐FRET signals were sequentially monitored at 37°C in a Flexstation 3 plate reader using the same settings as for binding experiments. Signal was expressed relative to the baseline TR‐FRET ratio established from the first three readings.

### 2.10 Endosomal FITC‐ligand binding assay

HEK293‐SNAP‐GLP‐1 receptor cells were labelled in suspension with SNAP‐Lumi4‐Tb (40 nM) for 1 h at room temperature in complete medium. Cells were then washed and resuspended in complete medium containing 100‐nM exendin‐4‐FITC, lixisenatide‐FITC, or no ligand, which was allowed to internalise over 30 min at 37°C. Cells were then placed on ice, washed with cold HBSS and incubated for 10 min in cold acetic acid +150‐mM NaCl buffer, pH 2.9, to strip surface ligand. After a final wash, cells were resuspended in HBSS and returned to 37°C. TR‐FRET signal was measured over 30 min in a Flexstation 3 plate reader using the same settings as for binding experiments.

### 2.11 TR‐FRET measurement of FITC‐ligand endocytosis

HEK293‐SNAP‐GLP‐1 receptor cells were labelled in suspension with SNAP‐Lumi4‐Tb (40 nM) for 1 h at room temperature in complete medium. After washing and resuspension in HBSS containing 0.1% BSA, cells were plated at 4°C to arrest endocytosis and pre‐chilled FITC‐ligands were allowed to bind for 3 h. The plate was then moved to the plate reader at 37°C to initiate endocytosis and TR‐FRET was measured at regular intervals using the same settings as for binding experiments. Ligand uptake was quantified by monitoring the change in TR‐FRET ratio over time relative to the baseline value from the average of the first two recordings.

### 2.12 Microscopes

#### 2.12.1 Confocal microscopy

Fixed, mounted coverslips were imaged with a Zeiss LSM780 inverted confocal microscope with a 63×/1.4 numerical aperture oil‐immersion objective. The same microscope with a water immersion 63×/1.2 numerical aperture objective in a 37°C heated chamber was used for RICS experiments.

#### 2.12.2 Widefield microscopy

Fixed, mounted coverslips were imaged at 20× or 40× using a widefield fluorescence microscope (Nikon Eclipse Ti2) with an LED light source.

#### 2.12.3 Electron microscopy

Resin‐embedded ultrathin 70 nm sections on copper grids were imaged on a FEI Tecnai T12 Spirit TEM. Images were acquired in a CCD camera (Eagle).

### 2.13 Raster image correlation spectroscopy (RICS)

HEK293‐SNAP‐GLP‐1 receptor cells were seeded onto poly‐d‐lysine‐coated MatTek dishes and surface‐labelled with SNAP‐Surface 488 (1 mM, 30 min at 37°C). After washing, cells were imaged at the basal plasma membrane in HBSS with 10‐mM HEPES at 37°C. SNAP‐Surface 488 was excited by a continuous wavelength laser at 488 nm and emission signal was collected at 500–580 nm. The pinhole was set to one Airy unit. Images of 256 × 256 pixels at 16‐bit depth were collected using 80 nm pixel size and 5 μs pixel dwell time for 200 consecutive frames at a single optical section. RICS analysis was performed using SimFCS 4 software (Globals Software, G‐SOFT Inc., Champaign, IL), as described elsewhere (Garcia & Bernardino de la Serna, 2018). To characterise the waist of the point spread function (PSF), 200 frames of freely diffusing recombinant EGFP (20 mM) were continuously collected. Analysis was performed on images where intensity trace was not decreased continuously by 20% or more over 50 frames to avoid possible bleaching artefacts that would interfere in diffusion coefficient measurements. A moving average (background subtraction) of 10 was applied, so that artefacts due to cellular motion or very slow‐moving particles were avoided. The obtained 2D autocorrelation map was then fitted, a surface map was obtained with the characterised PSF and the appropriate acquisition values for line time and pixel time. For different regions of interest (ROI) analyses within the same cell, the corresponding region was drawn employing a square region of 64 × 64 pixels.

### 2.14 Recycling measurements by widefield microscopy

HEK293‐SNAP‐GLP‐1 receptor cells seeded on coverslips were treated in serum‐free medium with agonist (100 nM) or vehicle for 30 min, in reverse time order, followed by washing and a variable recycling incubation period. Cells were then labelled using SNAP‐Surface‐549 (1 μM) in a complete medium for 30 min on ice to label surface receptor (contributed to by the recycled receptor population and any non‐internalised receptors during the agonist stimulation step), fixed with 4% paraformaldehyde and mounted in Diamond Prolong mounting medium. Slides were subsequently imaged by widefield microscopy. Five images were taken per coverslip in regions of high confluence using TRITC and FITC filter sets, followed by quantification of surface labelling in Fiji. Cell autofluorescence signal, estimated from a slide with cells that did not undergo SNAP‐labelling, was subtracted.

### 2.15 Recycling measurements by confocal microscopy

INS‐1832/3‐SNAP‐GLP‐1 receptor cells on coverslips were treated with agonist (100 nM) or vehicle for 30 min in serum‐free RPMI, followed by washing. Exendin‐4‐TMR (100 nM) was then added at the beginning of a 3‐h recycling period, after which cells were fixed with 4% paraformaldehyde, mounted in Diamond Prolong mounting medium with DAPI and three images acquired per coverslip by confocal microscopy. In this assay, total uptake of exendin‐4‐TMR is indicative of residual surface receptor at the end of exendin‐4/lixisenatide pretreatment (expected to be low) and the cumulative reappearance surface receptor during the recycling period. Quantification was performed in Fiji by expressing the integrated density of specific TMR signal relative to the number of cells (quantified by nuclei staining with DAPI) for each image.

### 2.16 Lysotracker co‐localisation assay

Surface SNAP‐GLP‐1 receptor was first labelled in INS‐1832/3‐SNAP‐GLP‐1 receptor cells with SNAP‐Surface‐488 (1 μM) for 30 min at 37°C. After washing, cells were treated in serum‐free RPMI, 11‐mM glucose, with agonist or vehicle added in reverse time order. Lysotracker‐DND99 (100 nM; Thermo Fisher) was added for the final 30 min before fixation and mounting as above. Co‐localisation of SNAP‐GLP‐1 receptor with Lysotracker was quantified from cell‐containing image regions as Mander's coefficient with auto‐threshold detection using the Coloc2 algorithm in Fiji. At least four images were analysed per coverslip.

### 2.17 Ultrastructural analysis of SNAP‐GLP‐1 receptor localisation by electron microscopy (EM)

INS‐1832/3‐SNAP‐GLP‐1 receptor cells on Thermanox coverslips (Agar Scientific) were labelled with 2‐μM SNAP‐Surface‐biotin (a gift from Dr Ivan Corrêa Jr, New England Biolabs), followed by 5 μg**·**ml^−1^ NaN_3_‐free Alexa Fluor 488 Streptavidin, 10‐nm colloidal gold (Molecular Probes) and stimulated with 100 nM of the indicated agonist for 1 h. Sample preparation for conventional EM was performed as previously described (Tomas, Futter, & Moss, 2004). Ultrathin 70 nm sections were cut *en face* with a diamond knife (DiATOME) and collected on 100 mesh hexagonal copper grids prior to imaging. Electron micrographs were individually thresholded to create binary images displaying only gold particles. All images were systematically processed to quantify gold‐labelled receptors so that multi‐particle aggregates would be registered as single large complexes as follows: First, the ImageJ “dilate” algorithm was applied three times so that adjacent gold particles within a complex would coalesce and second, the ImageJ “particle analysis” algorithm was run on the processed image to quantify the area of all particles and particle aggregates. The number of particles per aggregate was estimated using the determined size of clearly identified single gold particles after image processing. Forty images per treatment were quantified.

### 2.18 Insulin secretion assays

INS‐1832/3 cells were stimulated with agonist for 16–18 h in complete medium at 11‐mM glucose. At the end of the incubation period, a sample of supernatant was removed, diluted and analysed for secreted insulin by HTRF (Insulin High Range Assay, Cisbio). Insulin secretion was expressed relative to that from cells stimulated with 11‐mM glucose alone in the same experiment.

### 2.19 Animal studies

Animal studies are reported in compliance with the ARRIVE guidelines (Kilkenny et al., 2010) and with the recommendations made by the *British Journal of Pharmacology.* Mice are commonly used to test metabolic effects of GLP‐1 agonists (Greig et al., 1999). All animal procedures were approved by British Home Office under the UK Animal (Scientific Procedures) Act 1986 (Project Licence PB7CFFE7A). Lean male C57BL/6 mice (8–16 weeks of age, body weight 25–30 g, purchased from Charles River) were maintained at 21–23°C and light–dark cycles (12:12 h schedule, lights on at 07:00) in individually ventilated cages with woodchip bedding and a plastic tube plus shredded tissue paper for enrichment. Ad libitum access to water and normal chow diet (RM1, Special Diet Services) was provided unless otherwise stated. Mice were housed in groups of four, except for prior to food intake assessments when they were individually caged, with 1 week of acclimatisation prior to experiments. At the end of the study, mice were culled using CO_2_.

### 2.20 Intraperitoneal glucose tolerance tests

Mice were lightly fasted (2–3 h) before the test; 2 g·kg^−1^ of 20% glucose was injected via the i.p. route, either concurrently with agonist or after a specified delay. Blood glucose was recorded at the indicated time‐points from the tail vein using a handheld glucometer (GlucoRx Nexus).

### 2.21 Food intake assay

Individually caged mice were fasted overnight. Diet was returned immediately after a 100 μl i.p. injection of agonist or vehicle (0.9% NaCl) and intake monitored by measuring food weight at the indicated time‐points. Food spillage was not accounted for but would be expected to apply equally to each treatment.

### 2.22 Pharmacokinetic study

Mice were injected i.p. with 100 μl agonist and a 25 μl blood sample was subsequently taken from the tail vein into lithium‐heparin capillary tubes onto ice. The high ligand dose was required due to assay sensitivity. Plasma was separated and stored at −80°C prior to determination of exendin‐4/lixisenatide concentration using an ELISA which equally cross reacts with both peptides (exendin‐4 fluorescent EIA Kit, Phoenix Pharmaceuticals), with samples diluted 1:5 prior to analysis.

### 2.23 Group sizes, randomisation and blinding

#### 2.23.1 In vitro experiments

All in vitro experiments subjected to statistical analysis were performed with at least *n* = 5 biological replicates. Some experiments reported were repeated fewer than five times; examples include some corroborative confocal microscopy experiments performed to support quantitative DERET measures of internalisation, optimisation experiments, for example, for BG‐SS‐Lumi4‐Tb, or where the labour‐intensive nature of the experiments precluded multiple repeats, such as electron microscopy analyses. Such results should be considered exploratory. Throughout, one biological replicate was considered as the average of technical replicates (two to three) from each assay. Treatments were randomly distributed across microplates to avoid systematic bias. Each treatment to be compared was included in each experiment to allow matched analyses. Due to resource limitations, it was not possible to perform in vitro treatments in a blinded manner.

#### 2.23.2 In vivo experiments

All in vivo experiments included at least five mice per group. Group sizes were determined on the basis of previously established *n* numbers required to demonstrate the size of the effect expected from the in vitro results, without formal power calculations. Treatment order was randomly assigned and the average body weight within each group calculated to ensure this did not differ by more than 1 g. The investigator performing the experiment was blinded to treatment allocations.

### 2.24 Data and statistical analysis

TThe data and statistical analysis comply with the recommendations of the *British Journal of Pharmacology* on experimental design and analysis in pharmacology (Curtis et al., 2018). Quantitative data were analysed using Prism 8.0 (GraphPad Software). Mean ± standard error of mean (SEM), or individual replicates, are displayed throughout. Outliers were not removed prior to analysis. Normalisation was used for some in vitro assays to control for unwanted sources of variation between experimental repeats. Specifically, concentration–response data were expressed either relative to the maximal response of a reference compound measured as part of the same experiment or relative to vehicle‐treated control from the same experiment; nanoBiT recruitment responses, FRET biosensor responses and TR‐FRET internalisation and recycling kinetic responses were expressed relative to individual well baselines. Concentration–response data were analysed using four‐parameter logistic fits, with basal responses and Hill slopes globally constrained. As exendin‐4 and lixisenatide were both full agonists in all signalling assays, comparison of log EC_50_ values (relative potency ratios) was appropriate for determination of signal bias (Kenakin & Christopoulos, 2013); most figures refer to “pEC_50_” values, that is, modulus of log EC_50_, so that more potent ligands are assigned a higher value. Statistical comparisons were made by Student's *t*‐test or ANOVA as appropriate, either paired or matched depending on the experimental design. For analyses with >2 treatments, post hoc tests were only conducted if the overall ANOVA *P* value achieved statistical significance and there was no significant variance inhomogeneity. Tukey's test was used where multiple groups were compared to each other, and Sidak's test was used where two treatments were compared across multiple time‐points. The declared group size is the number of independent values and statistical analysis was done using these independent values (i.e., not treating technical replicates as independent values). Statistical significance was inferred if *P* < 0.05. Image analysis was performed in Fiji as above.

### 2.25 Nomenclature of targets and ligands

Key protein targets and ligands in this article are hyperlinked to corresponding entries in http://www.guidetopharmacology.org, the common portal for data from the IUPHAR/BPS Guide to PHARMACOLOGY (Harding et al., 2018) and are permanently archived in the Concise Guide to PHARMACOLOGY 2019/20 (Alexander et al., 2019).

## 3 RESULTS

### 3.1 Lixisenatide displays impaired coupling to intracellular cAMP signalling

**FIGURE 1:**
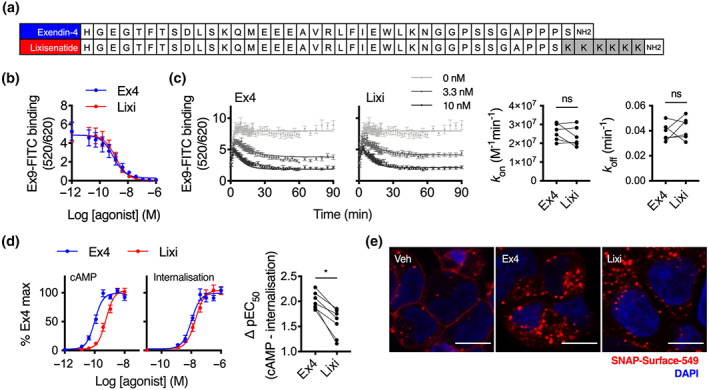
Lixisenatide displays selectively reduced coupling to cAMP signalling. (a) Sequences of each ligand in single amino acid code. (b) Equilibrium binding experiment using exendin‐4 (Ex4) or lixisenatide (Lixi) in competition with 10 nM exendin(9–39)‐FITC (Ex9‐FITC) in HEK293‐SNAP‐GLP‐1 receptor (R) cells, *n* = 5, see also Figure S1B. (c) Kinetic binding experiment using exendin‐4 or lixisenatide in competition with 10‐nM exendin(9–39)‐FITC in HEK293‐SNAP‐GLP‐1 receptor cells, with calculation of association (*k*_on_) and dissociation (*k*_off_) rate constants, *n* = 6, paired *t*‐tests. (d) Parallel measurements of cAMP production and GLP‐1 receptor internalisation in HEK293‐SNAP‐GLP‐1 receptor cells, 30‐min incubation, *n* = 7, four‐parameter fits of pooled data shown, bias analysis shows ΔpEC_50_ for cAMP‐internalisation responses, Student's paired *t*‐test. (e) SNAP‐GLP‐1 receptor endocytosis in HEK293‐SNAP‐GLP‐1 receptor cells labelled with SNAP‐Surface 549, 30‐min stimulation with 100‐nM ligand or vehicle (Veh), size bars: 8 μm, representative confocal images of *n* = 2 experiments. *P < 0.05 by statistical test indicated in the text. Data shown as mean ± SEM or individual replicates. n.s. not significant

The sequences of exendin‐4 and lixisenatide are shown in Figure 1a. We used a TR‐FRET approach (Emami‐Nemini et al., 2013) to measure binding of each ligand to human SNAP‐tagged GLP‐1 receptor in competition with a fluorescent GLP‐1 antagonist, exendin(9–39)‐FITC (Figure S1A–C). The equilibrium dissociation constants were similar for both agonists (log *K*_D_ −9.2 ± 0.1 vs. −9.0 ± 0.1 for exendin‐4 and lixisenatide, respectively, Figure 1b). Calculation of association and dissociation rate constants from kinetic binding experiments in competition with exendin(9–39)‐FITC also showed no differences between ligands (Figure 1c). However, in spite of similar binding affinities, cAMP signalling potency for lixisenatide was approximately five times lower than for exendin‐4 (log EC_50_–10.0 ± 0.1 vs. −9.3 ± 0.1 for exendin‐4 and lixisenatide respectively, Figure 1d). Potency for GLP‐1 receptor endocytosis, measured in parallel with the cAMP signalling experiments using diffusion‐enhanced resonance energy transfer (DERET), was also significantly reduced for lixisenatide, but to a more minor degree (log EC_50n_–8.0 vs. −7.7 ± 0.1 for exendin‐4 and lixisenatide respectively, see Figure S1D for kinetic traces), with analysis of pathway bias (scatter plot in Figure 1d) confirming that lixisenatide coupling to cAMP was selectively reduced by a factor of 3. Confocal imaging confirmed both ligands induced extensive SNAP‐GLP‐1 receptor endocytosis (Figure 1e).

As bias can be time‐point‐specific (Klein Herenbrink et al., 2016), we also used the FRET biosensor ^T^Epac^VV^ (Klarenbeek et al., 2011) to obtain real‐time readouts of cAMP signalling (Figure S1E). Comparison of ^T^Epac^VV^ and DERET potencies at 10‐min intervals indicated the selective loss of cAMP potency with lixisenatide was preserved throughout the stimulation period (Figure S1F). Downstream coupling to PKA activation was similarly reduced with lixisenatide (Figure S1G). These studies indicate that coupling of lixisenatide to cAMP signalling is reduced compared to with exendin‐4, in a manner unrelated to receptor occupancy.

### 3.2 Recruitment responses measured using NanoBiT complementation

**FIGURE 2:**
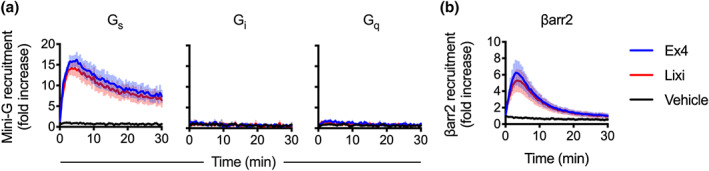
Recruitment of G proteins and β‐arrestin‐2 (ßarr) to GLP‐1 receptor. (a) Recruitment of LgBiT‐tagged mini‐G_s_, ‐G_i_ and ‐G_q_ to GLP‐1 receptor‐SmBit in transiently transfected HEK293T cells treated with 1‐μM exendin‐4 (Ex4) or lixisenatide (Lixi), measured by NanoBiT complementation, *n* = 5 (mini‐G_s_), *n* = 4 (mini‐G_i_) and *n* = 8 (mini‐G_q_). (b) As for (a) but recruitment of LgBiT‐tagged β‐arrestin‐2 (βarr2), *n* = 5. Data shown as mean ± SEM ß

To attempt to understand why lixisenatide shows reduced cAMP signalling despite similar binding affinity to exendin‐4, we used a NanoBiT complementation approach (Dixon et al., 2016) to measure recruitment of “mini‐G proteins” (Wan et al., 2018) and β‐arrestin‐2 to GLP‐1 receptor (Figure 2a,b). The GLP‐1 receptor construct developed in house for these assays, which bears a FLAG‐tag at the N‐terminus and a SmBiT tag at the C‐terminus, was validated by comparing GLP‐1‐induced cAMP responses in cells transiently transfected with the tagged versus non‐tagged GLP‐1 receptor, with similar cAMP potencies observed in each case (Figure S2A). We found that both ligands at a supramaximal concentration (1 μM) induced robust recruitment of mini‐G_s_, with a modestly reduced response with lixisenatide (vehicle‐subtracted AUC 300.1 ± 48.0 vs. 265.4 ± 47.6 for exendin‐4 and lixisenatide, respectively). Similar low amplitude mini‐G_q_ recruitment was detectable with each ligand (vehicle‐subtracted AUC 13.0 ± 3.0 vs. 7.9 ± 1.6 for exendin‐4 and lixisenatide, respectivel). By comparison, there was a robust mini‐G_q_ recruitment response to the SmBiT‐tagged endothelin A receptor when stimulated with endothelin‐1 (Figure S2B). Mini‐G_i_ recruitment following stimulation with either ligand was barely detectable. Recruitment of β‐arrestin‐2 was slightly reduced with lixisenatide compared to exendin‐4 (Figure 2b; vehicle‐subtracted AUC 58.3 ± 13.1 vs. 51.6 ± 13.8 for exendin‐4 and lixisenatide, respectively) Notably, β‐arrestin‐2 recruitment signal with both ligands showed more rapid decay than seen with mini‐G_s_, which is compatible with a previous report ascribing class A β‐arrestin kinetics to the GLP‐1 receptor (Al‐Sabah et al., 2014). To exclude luciferase substrate depletion as an explanation for the apparent rapid dissociation of β‐arrestin‐2, we performed control experiments in which the substrate was added at the end of the incubation, which showed the same pattern (Figure S2C). As the ligand concentration used was supramaximal, the implication of these studies is that lixisenatide is a modestly less efficacious ligand than exendin‐4 for both G_s_ and β‐arrestin‐2 recruitment.

### 3.3 GLP‐1 receptor clustering responses with exendin‐4 and lixisenatide

**FIGURE 3:**
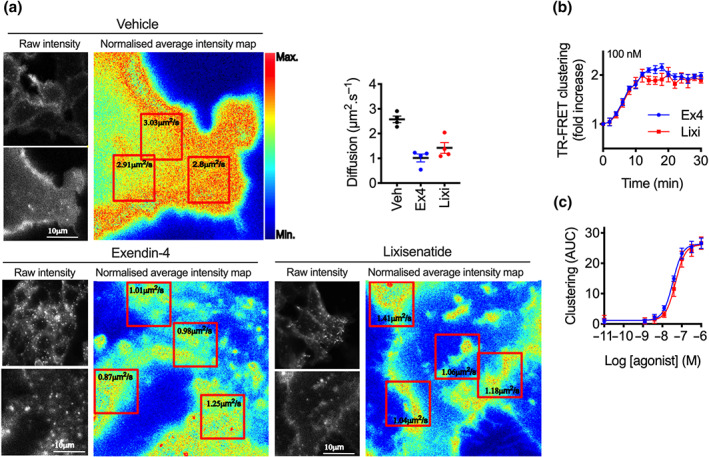
GLP‐1 receptor clustering with exendin‐4 (Ex4) and lixisenatide (Lixi). (a) Representative images from RICS analysis of GLP‐1 receptor clustering, showing SNAP‐Surface 488‐labelled GLP‐1 receptor imaged in the basolateral plane of HEK293‐SNAP‐GLP‐1 receptor cells after 10‐min treatment with vehicle (Veh) or 100‐nM agonist. Diffusion coefficients for individual ROIs are indicated on each image and average diffusion coefficient for each treatment are indicated from *n* = 4 experiments. (b) GLP‐1 receptor clustering kinetics measured by TR‐FRET in HEK293‐SNAP‐GLP‐1 receptor cells treated with 100 nM agonist, *n* = 5. (c) Concentration–response of GLP‐1 receptor clustering in HEK293‐SNAP‐GLP‐1 receptor cells, determined from AUC of kinetic responses, *n* = 5. *P < 0.05 by statistical test indicated in the text. Data shown as mean ± SEM or individual replicates

We previously demonstrated that GLP‐1 receptor segregates into plasma membrane nanodomains after stimulation with exendin‐4 and that this process is required for functional responses such as endocytosis (Buenaventura et al., 2019). As recent evidence raises the possibility that exendin‐4 simultaneously binds two GLP‐1 receptor protomers through a non‐canonical interaction made by its C‐terminus and the receptor extracellular domain (Koole et al., 2017), which might influence the receptor oligomeric state, we investigated whether the distinct C‐termini of exendin‐4 and lixisenatide could differentially modulate the GLP‐1 receptor clustering response. We employed raster image correlation spectroscopy (RICS), which has previously been used to demonstrate how ligand binding leads to receptor clusters with restricted diffusion in the plasma membrane (Compte et al., 2018). In HEK293‐SNAP‐GLP‐1 receptor imaged live, average diffusion coefficient was notably and similarly reduced by a factor of >2 after 10‐min treatment with both exendin‐4 and lixisenatide (Figure 3a and Figure S3A). We also used a dual labelling TR‐FRET method in which surface receptors were labelled with either Lumi4‐Tb (the donor) and SNAP‐Surface 649 (the acceptor), such that increased proximity of receptors results in an increase in signal (Buenaventura et al., 2019). Here, treatment with either ligand resulted in a similar TR‐FRET signal increase (Figures 3b,c and S3B), with no significant difference in potency across a full concentration range (log EC_50_ –7.5 ± 0.0 and −7.3 ± 0.1 for exendin‐4 and lixisenatide, respectively, *P* > 0.05 by Student's paired *t*‐test). Thus, we did not find evidence for differential GLP‐1 receptor clustering mediated by the distinct C‐termini of exendin‐4 versus lixisenatide.

### 3.4 Differences in GLP‐1 receptor recycling following exendin‐4 and lixisenatide treatment

**FIGURE 4:**
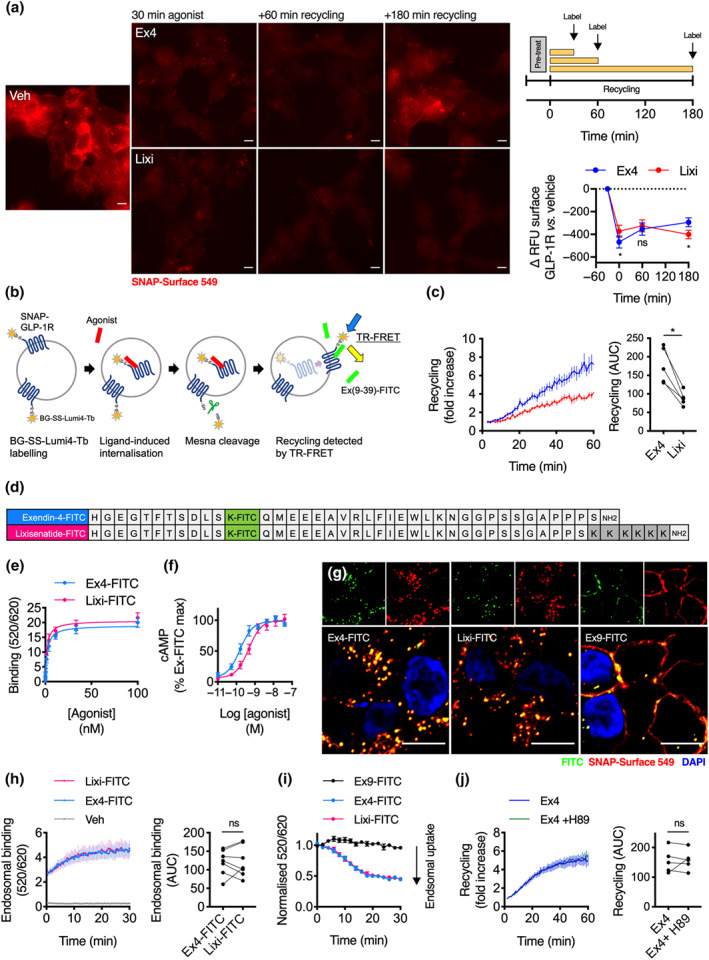
GLP‐1 receptor recycling with exendin‐4 (Ex4) versus lixisenatide (Lixi). (a) Non‐labelled HEK293‐SNAP‐GLP‐1 receptor (R) cells were treated with exendin‐4 or lixisenatide (100 nM) for 30 min, followed by washing, indicated recycling period and finally labelling of surface GLP‐1 receptor with SNAP‐Surface 549, representative microscopy images are shown with quantification of recycled receptor as relative fluorescence units (RFU) from *n* = 5 experiments, two‐way repeat measures ANOVA with Sidak's test, size bars: 8 μm. (b) Principle of TR‐FRET recycling assay. (c) GLP‐1 receptor recycling after exendin‐4 or lixisenatide treatment (100 nM, 30 min) in CHO‐K1‐SNAP‐GLP‐1 receptor cells, measured by TR‐FRET, *n* = 5, paired *t*‐test for AUC quantification. (d) Sequences of each FITC‐ligand in single amino acid code. (e) Saturation binding of exendin‐4‐FITC (Ex4‐FITC) and lixisenatide‐FITC (Lixi‐FITC) in HEK293‐SNAP‐GLP‐1 receptor cells, *n* = 6. (f) cAMP responses in HEK293‐SNAP‐GLP‐1 receptor cells, 30 min incubation, *n* = 5, four‐parameter fit shown. (g) Confocal microscopy images of HEK293‐SNAP‐GLP‐1 receptor cells labelled with SNAP‐Surface 549 and treated with indicated FITC‐ligand (100 nM) for 30 min, size bars: 8 μm, representative images of *n* = 2 independent experiments. (h) Endosomal binding of FITC‐ligands in HEK293‐SNAP‐GLP‐1 receptor cells, 100‐nM ligand treatment for 30 min prior to acid wash, *n* = 7, paired *t*‐test for AUC quantification. Note that the slight increase in signal over time is likely to represent restoration of “normal” endosomal pH following the prior exposure to pH 2.9. (i) TR‐FRET signal of FITC‐ligands in HEK293‐SNAP‐GLP‐1 receptor cells pre‐bound with indicated agonist (100 nM) at 4°C before initiating endocytosis by transfer to 37°C, normalised to initial TR‐FRET signal, *n* = 5. (j) GLP‐1 receptor recycling after exendin‐4 (100 nM, 30 min) treatment in CHO‐K1‐SNAP‐GLP‐1 receptor cells with or without prior treatment with H89 (10 μM), measured by TR‐FRET, *n* = 5, Student's paired *t*‐test for AUC quantification. *P < 0.05 by statistical test indicated in the text. Data shown as mean ± SEM or individual replicates

After initial endocytosis, differences in intracellular receptor trafficking can modulate GLP‐1 receptor‐induced insulin release (Buenaventura et al., 2018; Jones, Buenaventura, et al., 2018). To measure the rate of GLP‐1 receptor plasma membrane recycling, we treated HEK293‐SNAP‐GLP‐1 receptor cells with exendin‐4 or lixisenatide, followed by a variable recycling period, after which we applied a SNAP‐Surface fluorescent probe to label total surface GLP‐1 receptor. Treatment with lixisenatide versus exendin‐4 was associated with a slower rate of GLP‐1 receptor recycling (Figure 4a). To corroborate this finding, we developed a TR‐FRET assay to measure SNAP‐GLP‐1 receptor recycling in real‐time with a plate reader, using a cleavable form of the SNAP‐labelling TR‐FRET donor SNAP‐Lumi4‐Tb to allow reversible labelling by release of the fluorophore under mild reducing conditions (see Figure 4b for a graphical description of the assay principle and Figure S4A–F for further validation of the probe). Using CHO‐K1 cells stably expressing SNAP‐GLP‐1 receptor, which remain adherent during the multiple wash steps, we again found a reduced rate of GLP‐1 receptor recycling after lixisenatide compared to exendin‐4 treatment (Figure 4c).

As pH‐dependent dissociation of ligand–receptor complexes within acidic endosomes is known to influence post‐endocytic receptor sorting (Borden, Einstein, Gabel, & Maxfield, 1990), we wondered if the impact of low pH might specifically affect interactions made by the positively charged lixisenatide C‐terminus, modulating intra‐endosomal binding and thereby explaining its different recycling rate. We therefore developed FITC‐conjugates of each agonist (Figure 4d) to use as TR‐FRET acceptors in intra‐endosomal binding assays. Initial assessment showed that the FITC conjugate peptides recapitulated the pharmacological and trafficking properties of their unmodified counterparts, with similar binding affinity ( Figure 4e) but reduced cAMP signalling potency (Figure 3f) for lixisenatide‐FITC compared to exendin‐4‐FITC and extensive uptake for both ligands into GLP‐1 receptor‐containing endosomes (Figure 4g). In an acid wash assay (Figure S4G), TR‐FRET signals of each FITC‐ligand bound to internalised GLP‐1 receptor suggested that endosomal binding of each compound is similar (Figure 4h), arguing against our initial hypothesis. Moreover, the progressive loss of TR‐FRET signal from agonist pre‐bound to surface receptors at low temperature and subsequently endocytosed by return to 37°C was equal for both conjugates (Figures 4i and S4H, I), also suggesting intra‐endosomal dissociation of agonist‐receptor complexes does not differ between agonists. Overall, these results suggest the difference in recycling rate between exendin‐4 and lixisenatide is not related to differences in persistence of receptor binding within endosomes.

As GPCR recycling can be controlled by PKA (Vistein & Puthenveedu, 2013), we also wondered whether our observation of reduced PKA signalling despite similar occupancy for lixisenatide versus exendin‐4 (Figure S1G) might explain their different recycling rates. However, treatment with the PKA inhibitor H89 did not affect GLP‐1 receptor recycling after exendin‐4 pretreatment (Figure 4j).

### 3.5 Divergent effects of exendin‐4 and lixisenatide in pancreatic beta cells

**FIGURE 5:**
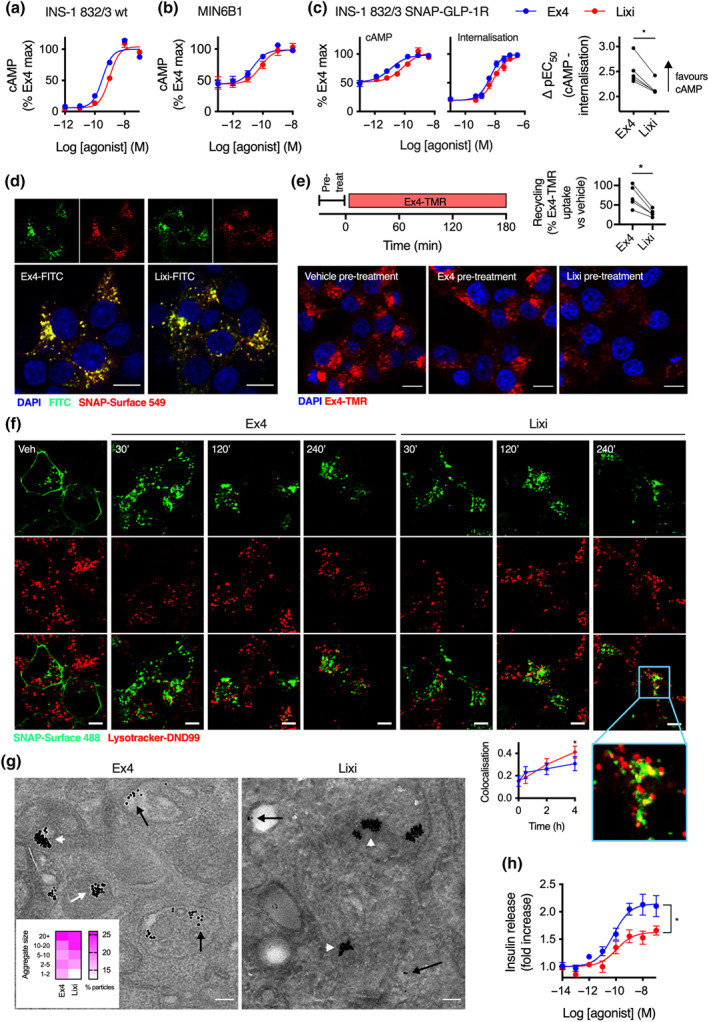
Effects of exendin‐4 (Ex4) and lixisenatide (Lixi) in beta cells. (a) Acute cAMP responses in wild type (wt) INS‐1832/3 cells treated with each ligand and 500 μM IBMX for 10 min, *n* = 6, four‐parameter fits of pooled data shown. (b) As for (a) but in MIN6B1 cells and *n* = 5. (c) Acute cAMP (30 min with 500 μM IBMX) and internalisation responses measured by DERET in INS‐1832/3‐SNAP‐GLP‐1 receptor (R) cells, *n* = 5, with quantification of bias and comparison by Student's paired *t*‐test, see also Figure S5A for DERET traces. (d) Confocal microscopy of INS‐1832/3‐SNAP‐GLP‐1 receptor cells labelled with SNAP‐Surface 549 and treated with indicated FITC‐ligand (100 nM) for 30 min, size bars: 8 μm, representative images of *n* = 2 independent experiments. (e) GLP‐1 receptor recycling measured in INS‐1832/3 SNAP‐GLP‐1 receptor cells treated with vehicle (Veh), exendin‐4 or lixisenatide (100 nM, 30 min) followed by washout and 3‐h exposure to exendin‐4‐TMR (Ex4‐TMR, 100 nM) during the recycling period, representative images from *n* = 5 experiments showing exendin‐4‐TMR uptake with quantification, Student's paired *t*‐test, size bars: 8 μm. (f) Confocal microscopy images of INS‐1832/3‐SNAP‐GLP‐1 receptor cells labelled with SNAP‐Surface 488 prior to stimulation with indicated agonist (100 nM), with Lysotracker‐DND99 added 15 min before the end of the incubation, representative images from *n* = 5 experiments with quantification by co‐localisation, two‐way repeat measures ANOVA with Sidak's test, size bars: 5 μm; inset, high magnification area. (g) Representative electron micrographs of INS‐1832/3‐SNAP‐GLP‐1 receptor cells labelled prior to stimulation with SNAP‐Surface‐biotin followed by 10 nm gold‐conjugated streptavidin and subsequently treated with exendin‐4 or lixisenatide (100 nM, 1 h), size bars: 0.1 μm; black arrows: single gold particles in endosomes, white arrows: lysosomal gold aggregates; quantification of size of aggregates from *n* = 40 images per treatment is shown on the heatmap. (h) Insulin secretion after 16‐h stimulation with exendin‐4 or lixisenatide in INS‐1832/3 cells, *n* = 7, four‐parameter fit shown, Student's paired *t*‐test to compare *E*_max_. *P < 0.05 by statistical test indicated in the text. Data shown as mean ± SEM or individual replicates

Potentiation of glucose‐stimulated insulin secretion is a major therapeutic goal of GLP‐1 antagonists treatment. We used rat insulinoma‐derived INS‐1832/3 beta cells to investigate differences between exendin‐4 and lixisenatide in the native cellular context for GLP‐1 receptor. As expected, reduced signalling potency for cAMP with lixisenatide was observed (Figure 5a) and also in mouse insulinoma‐derived MIN6B1 cells (Figure 5b). Moreover, the pattern of signal bias first demonstrated in HEK293 cells was recapitulated with SNAP‐GLP‐1 receptor ‐expressing INS‐1832/3 cells lacking endogenous GLP‐1 receptor (Naylor et al., 2016), with lixisenatide showing a relative preference for internalisation compared to cAMP signalling (Figures 5c and S5A). Similar uptake of exendin‐4‐FITC and lixisenatide‐FITC was observed by confocal microscopy (Figure 5d). To measure GLP‐1 receptor recycling after pretreatment with exendin‐4 or lixisenatide, we applied fluorescent exendin‐4‐TMR (Figure S5B, C) at the beginning of the recycling period following extensive wash of unlabelled agonist. As exendin‐4‐TMR is rapidly endocytosed by GLP‐1 receptors that reappear at the cell surface, its intracellular accumulation is indicative of the recycling rate. Again, recycling of GLP‐1 receptor after pretreatment with lixisenatide was less extensive than with exendin‐4 (Figure 5e).

Agonist‐internalised receptors which do not follow a recycling pathway can be sorted towards lysosomal degradation and in keeping with this, we found increased co‐localisation of SNAP‐GLP‐1 receptor with a fluorescent lysosomal marker after prolonged treatment with lixisenatide compared to exendin‐4 (Figure 5f). Moreover, electron microscopy imaging showed that, following live cell labelling of surface SNAP‐GLP‐1 receptors with a 10‐nm gold probe prior to agonist stimulation, the distribution of gold particles favoured larger size aggregates with lixisenatide versus exendin‐4 treatment (Figure 5g). This pattern of gold aggregation is indicative of probe target lysosomal degradation, as previously demonstrated for the EGF receptor (EGFR) (Futter & Hopkins, 1989; Futter, Pearse, Hewlett, & Hopkins, 1996). As excessive loss of surface GLP‐1 receptors without compensatory increases in recycling can limit insulinotropic efficacy (Jones, Buenaventura, et al., 2018), we measured cumulative insulin secretion after overnight treatment with each agonist (Figure 5H). Consistent with this paradigm, maximal insulin release with lixisenatide was reduced. Thus, the distinct pharmacological properties of lixisenatide and exendin‐4 translate to functional differences in beta cells.

### 3.6 Lixisenatide is less effective in vivo

**FIGURE 6:**
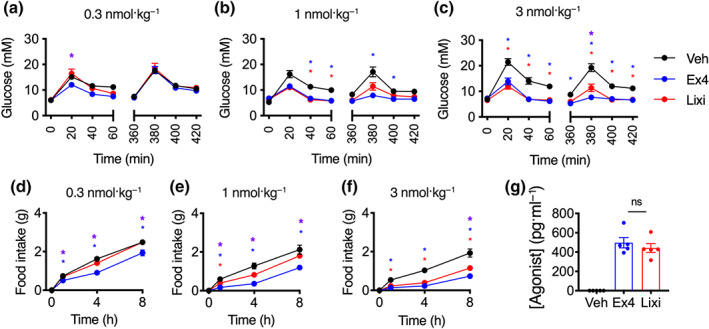
In vivo effects of exendin‐4 (Ex4) versus lixisenatide (Lixi). (a) i.p. glucose tolerance tests performed in lean mice at immediate and delayed (6‐h) time‐points after i.p. administration of 0.3 nmol·kg^−1^ ligand or vehicle (Veh; saline), 2 g·kg^−1^ glucose, *n* = 8 per group, two‐way repeat measures ANOVA with Tukey's test showing comparisons between exendin‐4 versus vehicle (blue asterisks), lixisenatide versus vehicle (red asterisks) and exendin‐4 versus lixisenatide (purple asterisks) at each time‐point. (b) As for (a) but with agonist dose of 1 nmol·kg^−1^. (c) As for (a) but with agonist dose of 3 nmol·kg^−1^ and *n* = 12 per group. (d) Cumulative food intake in overnight‐fasted lean mice after i.p. administration of 0.3 nmol·kg^−1^ ligand or vehicle (saline), *n* = 8 per group, two‐way repeat measures ANOVA with Tukey's test. (e) As for (d) but with agonist dose of 1 nmol·kg^−1^. (f) As for (d) but with agonist dose of 3 nmol·kg^−1^. (g) Agonist plasma concentrations in mice at 6 h after i.p. administration of indicated agonist (100 nmol·kg^−1^) or vehicle (saline), *n* = 5 per group, Student's unpaired *t*‐test. *P < 0.05 by statistical test indicated in the text. Data shown as mean ± SEM or individual replicates

GLP‐1 agonists are primarily used for the treatment of type 2 diabetes to reduce glycaemia and promote weight loss through appetite reduction. We assessed the glucoregulatory effects of each ligand at varying doses in mice via i.p. glucose tolerance tests performed immediately and 6 h after agonist treatment, to identify acute and delayed effects. We found that the anti‐hyperglycaemic effect of exendin‐4 was greater than equimolar lixisenatide (Figure 6a–c). Measurements of food intake in overnight‐fasted mice also showed that the anorectic effect of lixisenatide is reduced compared to exendin‐4 (Figure 6d–f). As expected from published data (Distiller & Ruus, 2008; Kolterman et al., 2005), these differences did not appear attributable to pharmacokinetics, as plasma concentrations of each agonist measured at 6 h after i.p. injection of a high dose of ligand (to ensure plasma levels remained within the detectable range) were similar (Figure 6g).

### 3.7 Biased agonists of lixisenatide display similar characteristics to their exendin‐4‐derived counterparts

**FIGURE 7:**
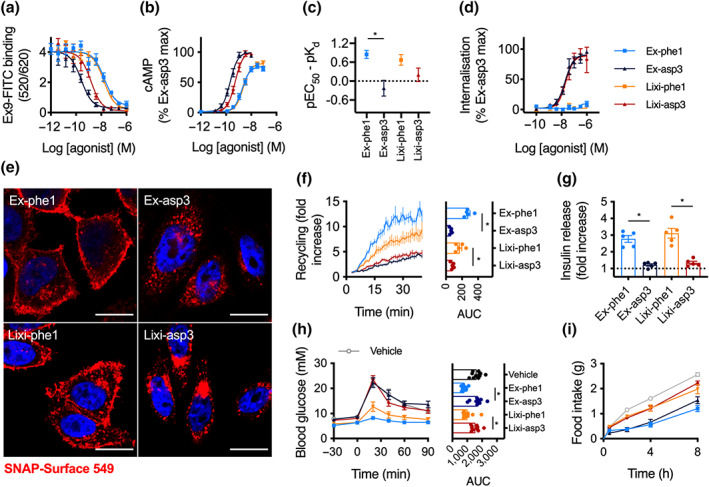
Comparison of biased exendin‐4 (Ex4) and lixisenatide (Lixi) analogues. (a) Equilibrium binding experiment using unlabelled agonists in competition with 10 nM exendin(9–39)‐FITC in HEK293‐SNAP‐GLP‐1 receptor (R) cells, *n* = 5, see also Figure S6B. (b) cAMP responses in HEK293‐SNAP‐GLP‐1 receptor cells, 30‐min incubation, *n* = 5, four‐parameter fits of pooled data shown. (c) Quantification of coupling of receptor occupancy to cAMP signalling for each ligand, quantified by subtracting pEC_50_ from data shown in (b) from pK_d_ shown in (a), with error propagation, one‐way ANOVA with Sidak's test. (d) GLP‐1 receptor endocytosis in HEK293‐SNAP‐GLP‐1 receptor cells measured by DERET, 30‐min incubation, *n* = 5, four‐parameter fits shown, see also Figure S6C. (e) Confocal microscopy images showing SNAP‐GLP‐1 receptor endocytosis in CHO‐K1‐SNAP‐GLP‐1 receptor cells labelled with SNAP‐Surface 549, 100‐nM agonist, 30‐min incubation, size bars: 12 μm, representative images from *n* = 2 experiments. (f) GLP‐1 receptor recycling in CHO‐K1‐SNAP‐GLP‐1 receptor cells, measured by TR‐FRET, 30‐min pretreatment with 100‐nM agonist followed by Mesna cleavage of surface BG‐SS‐Lumi4‐Tb and monitoring of recycling by TR‐FRET, *n* = 5, AUC compared by one‐way randomised block ANOVA with Tukey's test. (g) Insulin secretion in INS‐1832/3 cells treated at 11‐mM glucose ± indicated agonist (100 nM) for 16 h, expressed relative to response without agonist, *n* = 5, one‐way randomised block ANOVA with Tukey's test. (h) i.p. glucose tolerance tests performed in lean mice at delayed (8‐h) time‐points after i.p. administration of 2.4 nmol·kg^−1^ ligand or vehicle (Veh; saline), 2 g·kg^−1^ glucose, *n* = 10 (vehicle, exendin‐phe1, lixi‐asp3) or 11 (exendin‐asp3, lixi‐phe1) per group, one‐way ANOVA with Tukey's test comparing AUC for each treatment. (i) Cumulative food intake in overnight‐fasted lean mice injected i.p. with 2.4 nmol·kg^−1^ ligand or vehicle (saline), *n* = 12 per group. *P < 0.05 by statistical test indicated in the text. Data shown as mean ± SEM or individual replicates

Based on the observation that single amino acid substitutions close to the exendin‐4 N‐terminus can influence GLP‐1 receptor signalling and trafficking behaviours (Jones, Buenaventura, et al., 2018), we generated “lixi‐phe1” and “lixi‐asp3” (Figure S6A). In the context of exendin‐4, the ‐phe1 substitution is known to reduce binding affinity, reduce GLP‐1 receptor endocytosis, accelerate GLP‐1 receptor recycling, enhance insulin release and improve anti‐hyperglycaemic efficacy, while exendin‐asp3 shows opposing characteristics (Jones, Buenaventura, et al., 2018). Here, we found that, as for exendin‐phe1, lixi‐phe1 is a lower affinity ligand than its ‐asp3 counterpart (Figures 7a and Figure S6B). Similarly, acute cAMP signalling potency was reduced for both ‐phe1 variants (Figure 7b). However, when the reduced affinity was accounted for, exendin‐phe1 and, to a lesser extent, lixi‐phe1, were more efficiently coupled to cAMP production than the ‐asp3 versions (Figure 7c). Robust and similar plasma membrane clustering and endocytosis were induced by exendin‐asp3 and lixi‐asp3, but not either ‐phe1 peptide (Figures 7d and S6C, D). Note that some GLP‐1 receptor internalisation with ‐phe1 ligands was detectable by confocal microscopy in CHO‐K1‐SNAP‐GLP‐1 receptor cells (Figure 7e) and INS‐1 SNAP‐GLP‐1 receptor cells (Figure S6E). Recycling of endocytosed receptor was rapid with both ‐phe1 analogues (lixi‐phe1 less so than exendin‐phe1) and slow with the ‐asp3 equivalents (Figure 7f). The recycling rate correlated well with binding affinity when plotted on a log–log scale (Figure S6F). In INS‐1832/3 cells, insulin release after a 16‐h incubation at a maximal agonist concentration was notably higher with both ‐phe1 versus corresponding ‐asp3 ligands (Figure 7g), contrasting with the greater acute signalling potency with the latter in the same cell line (Figure S6G). Correspondingly, both ‐phe1 analogues exerted superior anti‐hyperglycaemic effects in an i.p. glucose tolerance tests performed 8 h after agonist administration, with exendin‐phe1 being the most effective (Figure 7h). The ‐asp3 peptides showed no effect in comparison to vehicle. In keeping with our previous observation that the trafficking effects of biased GLP‐1 agonists tend to exert a more pronounced effect on glucose regulation than on food intake (Jones, Buenaventura, et al., 2018), anorectic effects of ‐phe1 and ‐asp3 peptides were similar (Figure 7i), although the kinetics of the appetite suppressant effects were subtly different, with a greater proportion of the cumulative effect over 8 h achieved in the first 30 min with the ‐asp3 ligands, especially for exendin‐asp3 (Figure S6H).

Two main conclusions can be made from this set of experiments: (a) Sequence substitutions close to the N‐termini of both parent peptides consistently result in a linked set of in vitro and in vivo agonist characteristics with the potential to improve their therapeutic properties and (b) this effect is most marked for exendin‐4, for which the differences between ‐phe1 and ‐asp3 analogues was generally greater than for lixisenatide.

## 4 DISCUSSION

In this study, we performed a side‐by‐side pharmacological evaluation of two closely related GLP‐1 , receptor agonists both of which are in routine clinical usage and differ structurally only by the hexalysine C‐terminal extension in lixisenatide. We found that, despite similar binding affinity, coupling of lixisenatide to cAMP signalling was reduced compared to exendin‐4. After similar levels of GLP‐1 receptor plasma membrane clustering and endocytosis induced by both peptides, recycling of GLP‐1 receptor was slower after lixisenatide treatment, with apparent preferential targeting to a degradative lysosomal pathway. This resulted in a reduction in insulinotropic efficacy and the ability to control blood glucose 6–8 h after dosing in mice. Therefore, the structural differences between the C‐termini of each peptide appear to have some functional importance. See Table 1 for a summary of the properties of each ligand.

**TABLE 1:** Summary of pharmacological properties of exendin‐4 and lixisenatide

Readout	Cell model	Exendin‐4	Lixisenatide
Affinity	HEK293‐SNAP‐GLP‐1R	++	++
cAMP	HEK293‐SNAP‐GLP‐1R	++	+
	INS‐1832/3	++	+
	MIN6B1	++	+
Mini‐G_s_ recruitment	HEK293T	++	+
βarr2 recruitment	HEK293T	++	+
Plasma membrane clustering	HEK293‐SNAP‐GLP‐1R	++	++
Endocytosis	HEK293‐SNAP‐GLP‐1R	++	++
	INS‐1832/3‐SNAP‐GLP‐1R	++	++
Recycling	HEK293‐SNAP‐GLP‐1R	++	+
	INS‐1832/3‐SNAP‐GLP‐1R	++	+
Lysosomal targeting	INS‐1832/3‐SNAP‐GLP‐1R	+	++
Insulin secretion	INS‐1832/3	++	+

Our finding here of reduced cAMP signalling potency with lixisenatide matches our earlier observation (Jones, Buenaventura, et al., 2018). We also found reduced cAMP potency in rat INS‐1832/3 and mouse MIN6B1 cells, arguing against a species‐specific effect. Unfortunately, in the absence of a high level of confidence of the position of the exendin‐4 C‐terminus in published GLP‐1 receptor structural studies (Liang et al., 2018), a robust molecular explanation for the signalling deficit of lixisenatide remains elusive. Runge et al. found the exendin‐4 Trp‐cage does not directly interact with the GLP‐1 receptor extracellular domain (Runge, Thøgersen, Madsen, Lau, & Rudolph, 2008), although analysis of photo‐crosslinking data using the full length receptor showed that the flexible exendin‐4 C‐terminus might snake over the top of the extracellular domain and form interactions with Phe80, Tyr101 and Phe103 (Koole et al., 2017). Ligand‐specific C‐termini could plausibly alter this interaction, with resultant changes to the orientation of the ligand N‐termini and knock‐on effects for receptor activation. Alternatively, the Trp‐cage might act in trans across GLP‐1 receptor homodimers (Koole et al., 2017), resulting in changes in its oligomeric state, a factor known to influence coupling to signalling intermediates (Milligan, Ward, & Marsango, 2019), including for GLP‐1 receptor (Buenaventura et al., 2019; Harikumar et al., 2012). However, we observed no major differences between the GLP‐1 receptor clustering responses induced by either ligand. Using NanoBiT complementation, we found subtle differences in GLP‐1 receptor coupling to both G_s_ and β‐arrestin‐2 between exendin‐4 and lixisenatide, although it is not clear whether this is sufficient to explain the significant differences in potency for cAMP signalling. In our assays, we found that each ligand induced only minor levels of recruitment of mini‐G_q_ to GLP‐1 receptor, raising questions about the importance of signalling via this G protein in GLP‐1 receptor responses (Shigeto et al., 2015). However, we cannot exclude cell type‐specific effects, or the possibility that the recruitment pattern of catalytically inactive mini‐G_q_ underestimates the potential for G_q_ activation.

The other key finding here pertains to the post‐endocytic trafficking of the two ligands, which was evaluated across different cell systems using a variety of complementary approaches. Despite similar internalisation profiles, GLP‐1 receptor recycling after lixisenatide treatment was slower in both HEK293 and INS‐1832/3 beta cells. In contrast, a higher degree of lixisenatide‐stimulated GLP‐1 receptors tended to progressively co‐localise with the lysosomotropic fluorescent probe Lysotracker, indicating preferential targeting of the receptor towards a degradative pathway. This was in agreement with the increased level of intracellular gold‐conjugated SNAP‐tag probe aggregation detected by electron microscopy, suggesting enhanced tendency for lysosomal degradation of the lixisenatide‐stimulated receptor. These phenotypes partly recapitulate the differences previously observed with biased exendin‐4‐derived GLP‐1 agonists (Jones, Buenaventura, et al., 2018), which were linked to diminished insulin secretion efficacy. Indeed, we found in the present study that maximal insulin secretion was reduced when beta cells were exposed to lixisenatide versus exendin‐4 over a sustained exposure period. However, despite these broadly similar sets of findings, the mechanism for slowing of GLP‐1 receptor recycling with lixisenatide did not appear to depend on greater binding affinity, a factor that was previously demonstrated to influence the recycling of biased exendin‐4‐derived GLP‐1 receptor agonists (Jones, Buenaventura, et al., 2018). We specifically aimed to address the possibility of protonation of the hexalysine scaffold of lixisenatide in acidic conditions leading to altered intra‐endosomal agonist dissociation but could find no evidence for this. We did not examine post‐translational modifications linked to target degradation such as ubiquitination (Clague & Urbé, 2010), but note that the GLP‐1 receptor is not ubiquitinated by treatment with exendin‐4, despite a considerable amount of receptor degradation measurable after continuous exposure to this agonist (Jones, Bloom, et al., 2018). The reason for the increased lysosomal post‐endocytic targeting of the GLP‐1 receptor with lixisenatide therefore remains unclear. It should also be emphasised that we only measured recycling after stimulation with a high ligand concentration so as to promote a large degree of initial GLP‐1 receptor endocytosis and we cannot be certain that the same effects would be observed at the lower concentrations likely to be encountered in vivo.

Our observations of generally reduced biological effect of lixisenatide for physiologically important readouts suggest that these pharmacological differences are indeed translated to differences in downstream responses. In particular, we found reduced efficacy for sustained insulin secretion with lixisenatide using an in vitro beta cell system, as well as reduced anti‐hyperglycaemic and anorectic effects in mice. This observation comes with the caveat that we cannot be certain that the effects observed in vivo represent the same phenomena as observed with our prolonged in vitro incubations. Moreover, as our studies were performed only in male mice, we cannot exclude the possibility of sex‐specific effects. As the “advantages” of exendin‐4 in vivo were detectable acutely, in a dose‐dependent manner, it is likely that they are partly attributable to agonist potency differences rather than the post‐endocytic trafficking phenotypes. However, the potential for GLP‐1 receptor endocytosis to influence pharmacodynamics of exendin‐4 has previously been modelled (Gao & Jusko, 2012) and while this model focused on receptor internalisation, differences in recycling and degradation rates could plausibly be linked via similar mechanisms.

Differentiation in the therapeutic profiles of GLP‐1 antagonists in humans has been noted on many occasions (Aroda, 2018). A head‐to‐head comparison in patients with type 2 diabetes showed numerically greater HbA1c reduction and weight loss with exenatide compared to lixisenatide (Rosenstock et al., 2013). Exenatide showed a beneficial effect on cardiovascular outcomes, albeit with borderline significance (Holman et al., 2017), whereas a separate trial of lixisenatide found no evidence of benefit (Pfeffer et al., 2015). However, understanding the link between the receptor pharmacology observed in our study and real‐world performance of each agonist is hampered by the different dosing and administration schedules (10 μg twice daily for exenatide, or weekly as a sustained release preparation, compared to 20 μg once daily for lixisenatide).

Following the distinctive effects, we previously observed with biased GLP‐1 antagonists derived from exendin‐4 (Jones, Buenaventura, et al., 2018), we developed biased lixisenatide‐derived compounds based on a similar design. While we did not compare these against their parent ligands, the ‐phe1 substitution in both exendin and lixisenatide configurations displayed favourable characteristics such as reduced internalisation and fast recycling compared to the ‐asp3 variants. This translated to improved insulin secretion in vitro and significantly better anti‐hyperglycaemic effect in vivo. These observations therefore add to the evidence that modifications to GLP‐1 antagonist N‐termini are capable of inducing functionally important signal bias (Zhang et al., 2015; Jones, Buenaventura, et al., 2018; Fremaux et al., 2019).

In summary, our study provides insights into specific signalling and trafficking differences of two GLP‐1 antagonists in routine clinical use, linking these characteristics to their effects in vivo. The precise molecular mechanisms underpinning these differences remains to be elucidated.

## AUTHOR CONTRIBUTIONS

P. P., M. L., Z. F., A. T., B. J., J. B. d. l. S designed the study. P. P., M. L., Z. F., A. T., B. J., S. B., J. B. d. l. S performed and analysed experiments. J. B. and D. J. H. provided novel reagents. A. T., B. J., G. A. R., S. R. B. and J. M acquired funding. B. J., A. T. and P. P. wrote the manuscript. All authors reviewed and approved the manuscript.

## CONFLICT OF INTEREST

G. A. R. is a consultant for Sun Pharmaceuticals and has received grant funding from Sun Pharmaceuticals and Les Laboratoires Servier. B. J. and A. T. have received grant funding from Sun Pharmaceuticals.

## DECLARATION OF TRANSPARENCY AND SCIENTIFIC RIGOUR

This Declaration acknowledges that this paper adheres to the principles for transparent reporting and scientific rigour of preclinical research as stated in the *BJP* guidelines for Design and Analysis and Animal Experimentation, and as recommended by funding agencies, publishers and other organisations engaged with supporting research.

## Supporting information


**Figure S1.** (**A**) Sequence of exendin(9–39)‐FITC in single amino acid code. (**B**) Saturation binding of exendin(9–39)‐FITC in HEK293‐SNAP‐GLP‐1R cells, *n* = 5, performed in parallel with experiments shown in Figure 1B. (**C**) Confocal microscopy images showing specific exendin(9–39)‐FITC binding (100 nM, 30 minutes) to SNAP‐Surface 549‐labelled INS‐1832/3 SNAP‐GLP‐1R cells with or without prior treatment with exendin‐4 (10 μM). (**D**) DERET internalisation traces with indicated concentration of agonist in HEK293‐SNAP‐GLP‐1R cells, *n* = 7, relates to Figure 1D. (**E**) cAMP responses measured using TEpacVV biosensor in HEK293‐SNAP‐GLP‐1R cells with indicated concentration of agonist, normalized to individual well baselines, *n* = 5. (**F**) Analysis of bias at 3 timepoints from (D) and (E), with dose responses constructed from averaged data across all experimental repeats split into 10‐min bins to derive single values for pEC50, lixisenatide responses were subtracted from those of exendin‐4 at each time‐point. (**G**) Cytoplasmic PKA signalling in CHO‐K1‐SNAP‐GLP‐ 1R cells stably expressing AKAR4‐NES biosensor stimulated with indicated concentration of agonist, *n* = 5, 4‐parameter fit of AUC shown with pEC50 values compared by paired *t*‐test. *P < 0.05 by statistical test indicated in the text. Data indicated as mean ± SEM.
