# Supplementary material for: Signalling, trafficking and glucoregulatory properties of glucagon‐like peptide‐1 receptor agonists exendin‐4 and lixisenatide
Source: Br J Pharmacol. 2020 Jun 19;177(17):3905–23. doi: 10.1111/bph.15134 (PMC7429481; doi:10.1111/bph.15134)
Supplement: Supplementary file 1 — Figure S1. (A) Sequence of exendin(9–39)‐FITC in single amino acid code. (B) Saturation binding of exendin(9–39)‐FITC in HEK293‐SNAP‐GLP‐1R cells, n = 5, performed in parallel with experiments shown in Figure 1B. (C) Confocal microscopy images showing specific exendin(9–39)‐FITC binding (100 nM, 30 minutes) to SNAP‐Surface 549‐labelled INS‐1832/3 SNAP‐GLP‐1R cells with or without prior treatment with exendin‐4 (10 μM). (D) DERET internalisation traces with indicated concentration of agonist in HEK293‐SNAP‐GLP‐1R cells, n = 7, relates to Figure 1D. (E) cAMP responses measured using TEpacVV biosensor in HEK293‐SNAP‐GLP‐1R cells with indicated concentration of agonist, normalized to individual well baselines, n = 5. (F) Analysis of bias at 3 timepoints from (D) and (E), with dose responses constructed from averaged data across all experimental repeats split into 10‐min bins to derive single values for pEC50, lixisenatide responses were subtracted from those of exendin‐4 at each time‐point. (G) Cytoplasmic PKA signalling in CHO‐K1‐SNAP‐GLP‐ 1R cells stably expressing AKAR4‐NES biosensor stimulated with indicated concentration of agonist, n = 5, 4‐parameter fit of AUC shown with pEC50 values compared by paired t‐test. *P < 0.05 by statistical test indicated in the text. Data indicated as mean ± SEM. [file BPH-177-3905-s001.pdf]

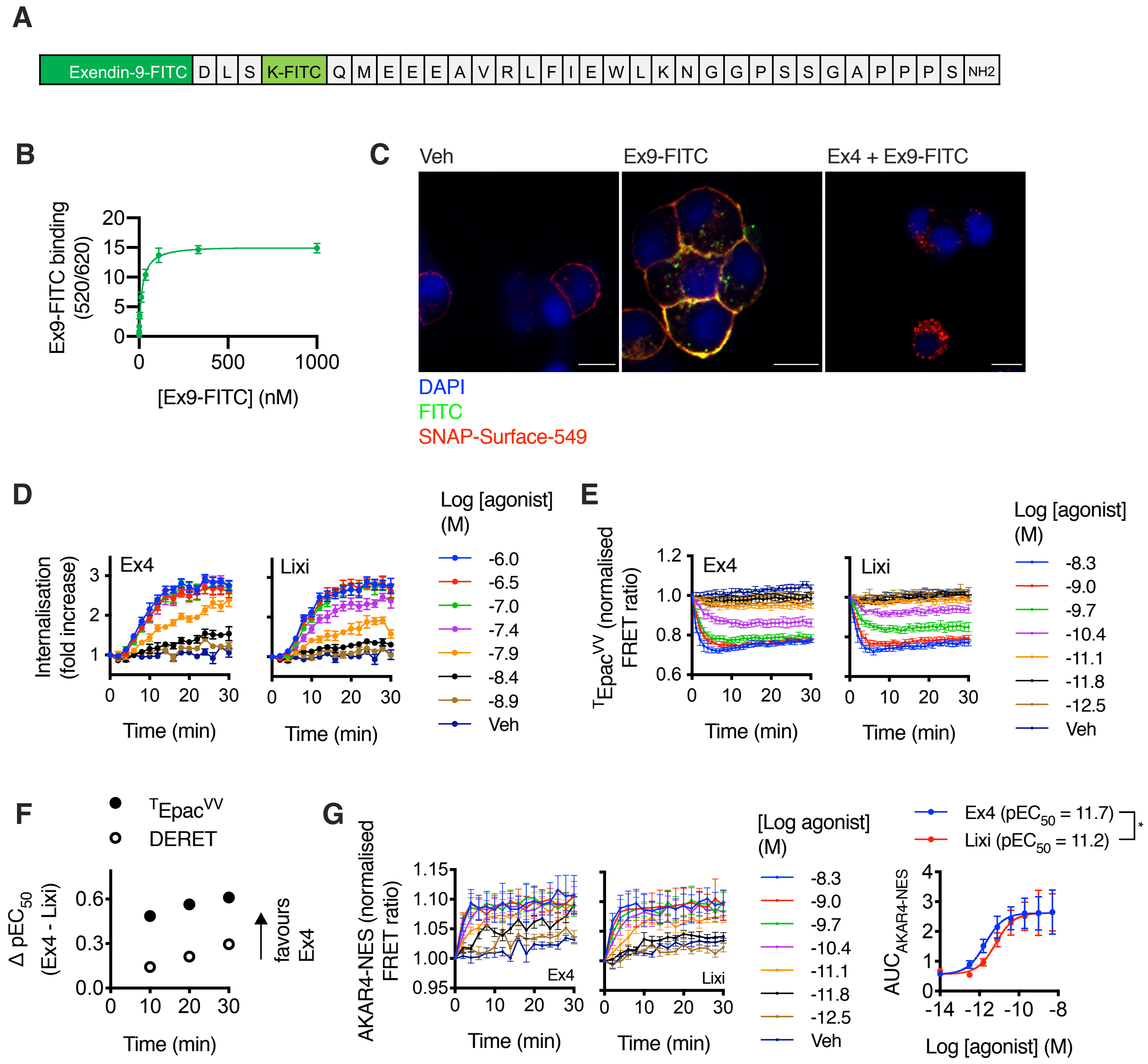

Supplementary Figure 1. (**A**) Sequence of exendin(9-39)-FITC in single amino acid code. (**B**) Saturation binding of exendin(9-39)-FITC in HEK293-SNAP-GLP-1R cells, *n*=5, performed in parallel with experiments shown in Figure 1B. (**C**) Confocal microscopy images showing specific exendin(9-39)-FITC binding (100 nM, 30 minutes) to SNAP-Surface 549-labelled INS-1 832/3 SNAP-GLP-1R cells with or without prior treatment with exendin-4 (10 μM). (**D**) DERET internalisation traces with indicated concentration of agonist in HEK293-SNAP-GLP-1R cells, *n*=7, relates to Figure 1D. (**E**) cAMP responses measured using $^{T}Epac^{VV}$ biosensor in HEK293-SNAP-GLP-1R cells with indicated concentration of agonist, normalized to individual well baselines, *n*=5. (**F**) Analysis of bias at 3 time-points from (D) and (E), with dose responses constructed from averaged data across all experimental repeats split into 10-min bins to derive single values for pEC_{50}, lixisenatide responses were subtracted from those of exendin-4 at each time-point. (**G**) Cytoplasmic PKA signalling in CHO-K1-SNAP-GLP-1R cells stably expressing AKAR4-NES biosensor stimulated with indicated concentration of agonist, *n*=5, 4-parameter fit of AUC shown with pEC_{50} values compared by paired t-test. *p<0.05 by statistical test indicated in the text. Data indicated as mean ± SEM.

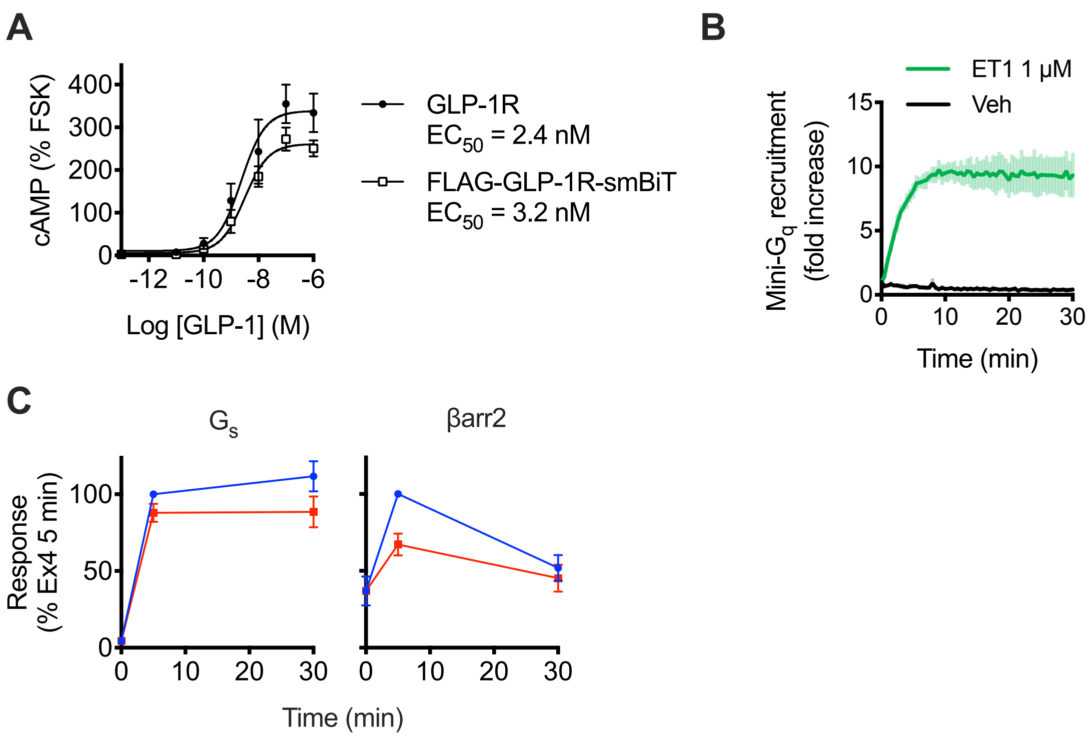

Supplementary Figure 2. (**A**) cAMP signalling potency of untagged GLP-1R and FLAG-GLP-1R-smBiT tagged GLP-1R expressed transiently in HEK293T cells, 30-min stimulation without IBMX, normalised to repsonse obtained with 10 µM forskolin (FSK), *n*=3. (**B**) Mini-G_q recruitment to endothelin A receptor transiently expressed in HEK293T cells and stimulated with vehicle (Veh) or endothelin-1 (ET1), *n*=4. (**C**) NanoBiT measurement of miniG_s and β-arrestin-2 recruitment to GLP-1R in endpoint mode, with fumarizine substrate added 2-min before indicated timepoint.

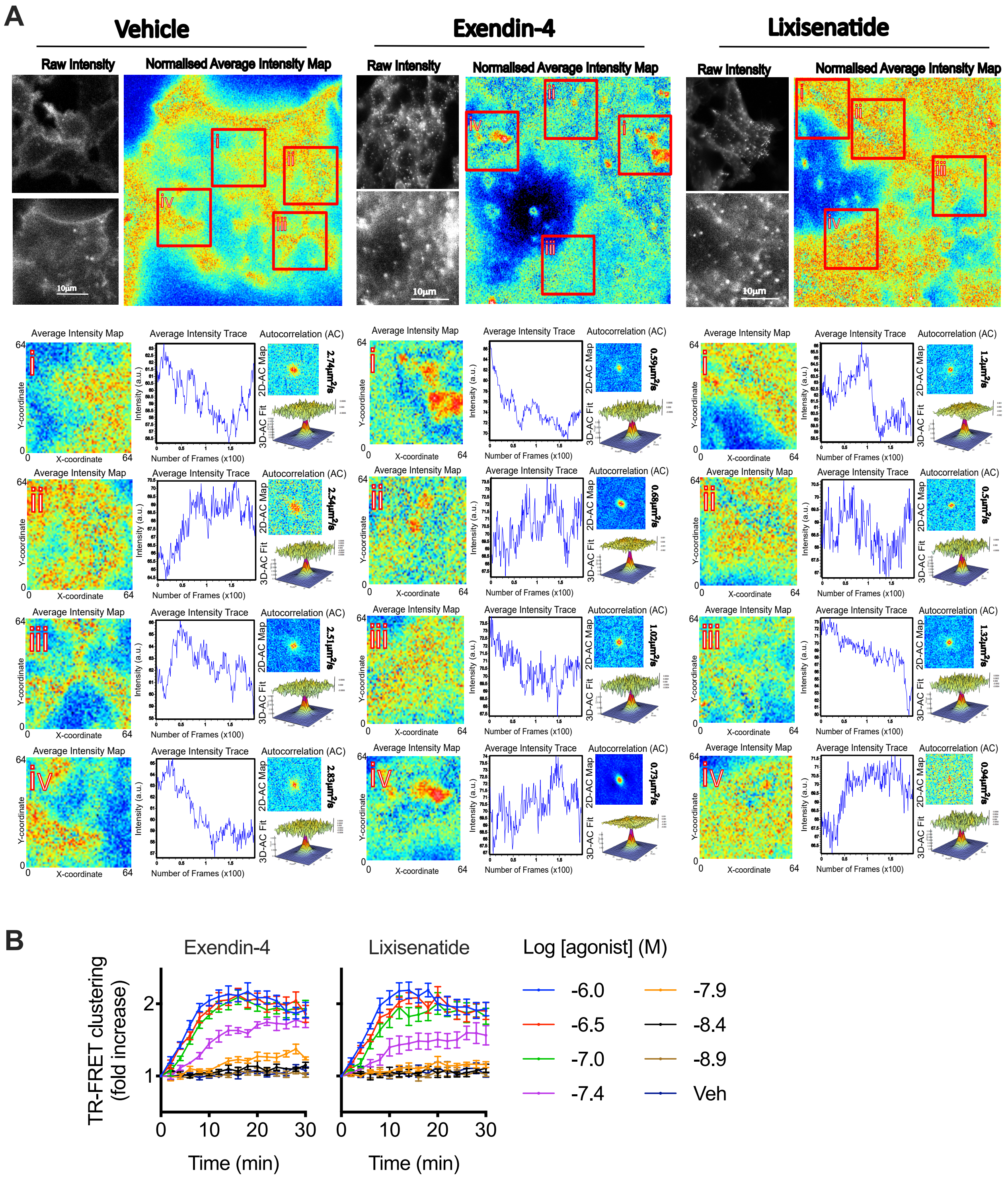

Supplementary Figure 3. (**A**) Additional images acquired for GLP-1R clustering measured by RICS, including average intensity traces and 2D autocorrelation maps with 3D fitting, as described in methods section. (**B**) Kinetic traces showing GLP-1R clustering measured by TR-FRET in HEK293-SNAP-GLP-1R cells, *n*=5. Data shown as mean ± SEM.

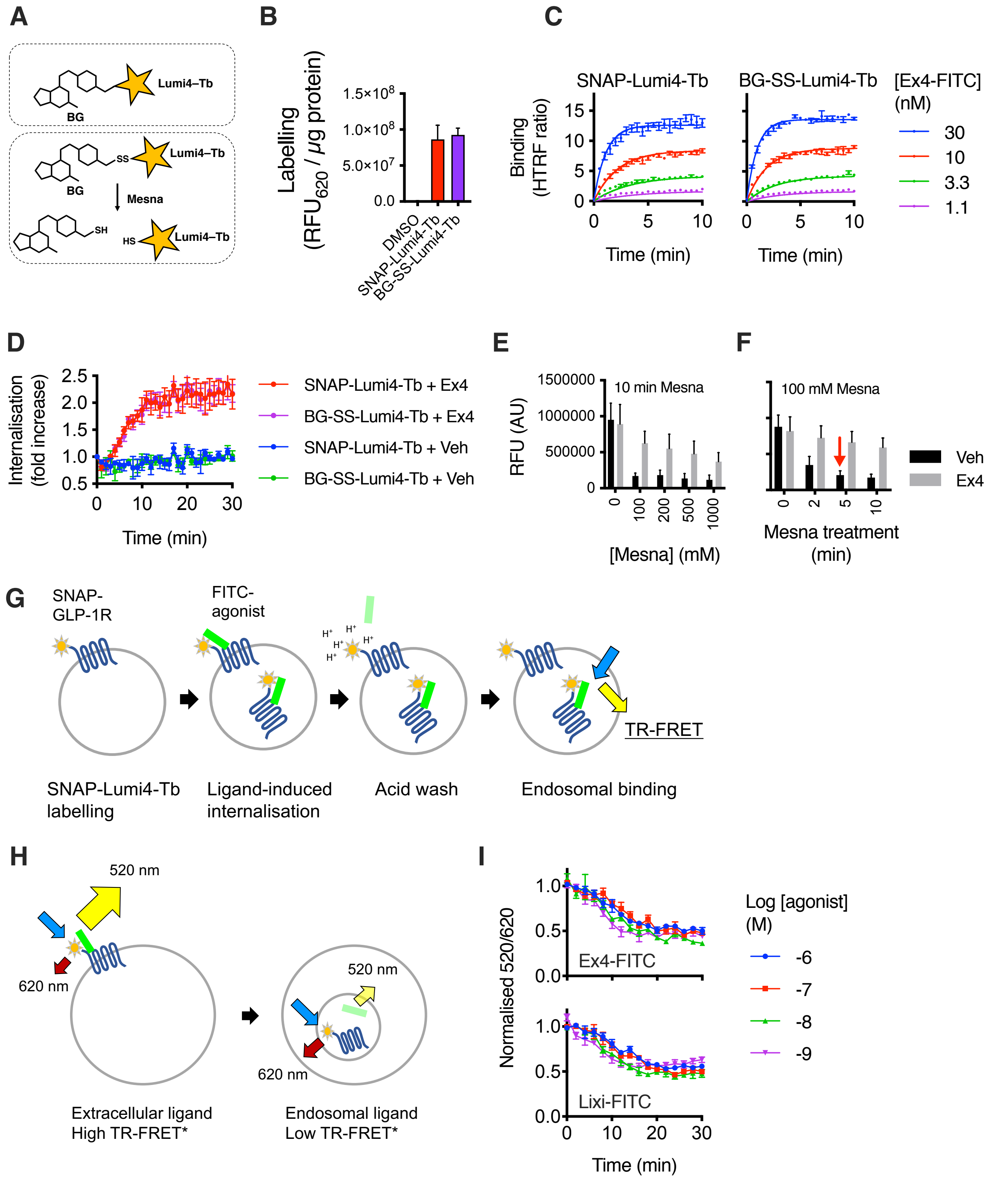

Supplementary Figure 4. (**A**) Cartoon depicting cleavage of BG-SS-Lumi4-Tb using Mesna. (**B**) Labelling efficiency of SNAP-Lumi4-Tb and BG-SS-Lumi4-Tb (both 40 nM) in HEK293-SNAP-GLP-1R cells, normalised to protein content, *n*=2. (**C**) Kinetic binding of exendin-4-FITC in HEK293-SNAP-GLP-1R cells labelled with SNAP-Lumi4-Tb or BG-SS-Lumi4-Tb, *n*=3. (**D**) Exendin-4-induced GLP-1R internalisation measured by DERET in HEK293-SNAP-GLP-1R cells labelled with SNAP-Lumi4-Tb or BG-SS-Lumi4-Tb, *n*=3. (**E**) Optimisation of Mesna cleavage of surface SNAP-GLP-1R in CHO-K1-SNAP-GLP-1R cells labelled with BG-SS-Lumi4-Tb and treated ± exendin-4 (100 nM, 30 minutes) before treatment with Mesna at indicated concentration for 10 minutes at 4°C, *n*=2. (**F**) As for (E) but with fixed Mesna dose for indicated incubation period; red arrow highlights how maximum cleavage

was achieved by 5 minutes. (**G**) Principle of endosomal binding experiment shown in Figure 3H. (**H**) Principle of FITC-ligand uptake experiment shown in Figure 3I. (**I**) FITC-ligand uptake in HEK293-SNAP-GLP-1R cells pre-bound with indicated ligand and dose at 4°C before triggering endocytosis by transfer to 37°C, *n*=3. Data indicated as mean ± SEM.

A

B

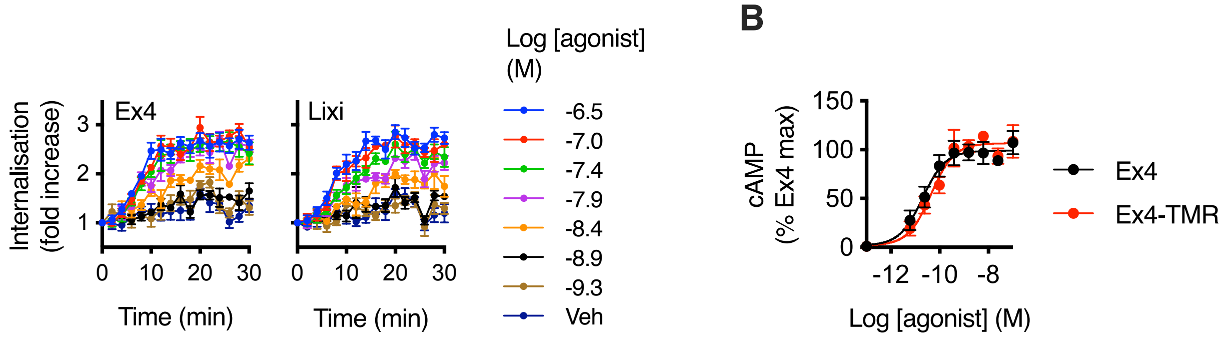

C

Exendin-4	H	G	E	G	T	F	T	S	D	L	S	K	Q	M	E	E	E	A	V	R	L	F	I	E	W	L	K	N	G	G	P	S	S	G	A	P	P	P	S	NH2
Exendin-4-TMR	H	G	E	G	T	F	T	S	D	L	S	K-TMR	Q	M	E	E	E	A	V	R	L	F	I	E	W	L	K	N	G	G	P	S	S	G	A	P	P	S	S	NH2

Supplementary Figure 5. (**A**) DERET internalisation traces with indicated concentration of agonist in INS-1 832/3-SNAP-GLP-1R cells, *n*=5, relates to Figure 5C. (**B**) cAMP responses of exendin-4 and exendin-4-TMR in HEK293-SNAP-GLP-1R cells, 30-min stimulation, 4-parameter fits of pooled data shown, *n*=4. (**C**) Amino acid sequence of exendin-4 and exendin-4-TMR in single letter amino acid code. Data indicated as mean ± SEM.

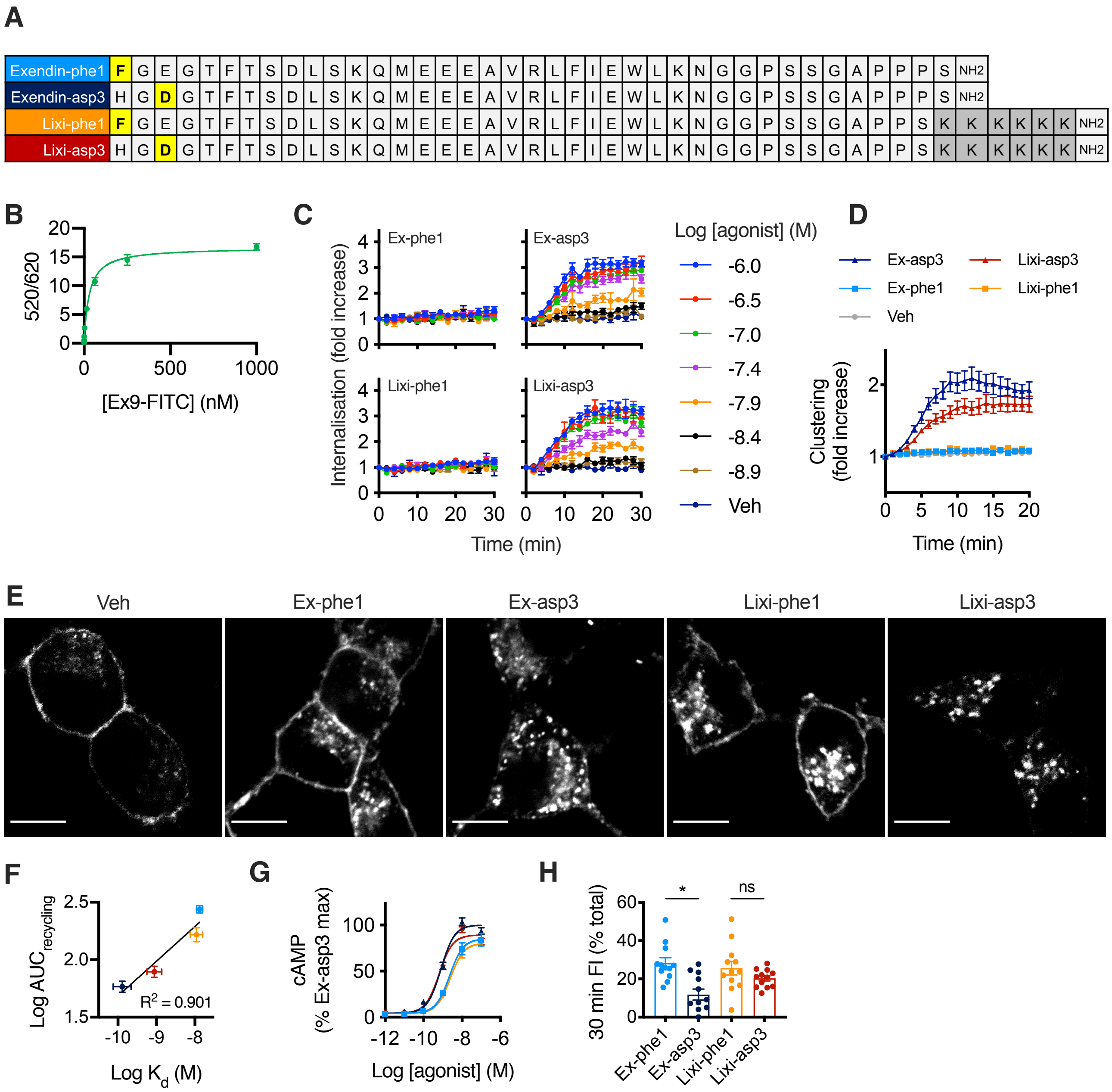

Supplementary Figure 6. (**A**) Peptide sequences of biased exendin-4 and lixisenatide analogues in single letter amino acid code. (**B**) Saturation binding of exendin(9-39)-FITC in HEK293-SNAP-GLP-1R cells, *n*=5, performed in parallel with experiment shown in Figure 7A. (**C**) DERET internalisation traces with indicated concentration of agonist in HEK293-SNAP-GLP-1R cells, *n*=5, relates to Figure 7D. (**D**) GLP-1R clustering responses induced by 100 nM agonist in HEK293-SNAP-GLP-1R cells, *n*=5. (**E**) Confocal microscopy images showing SNAP-GLP-1R endocytosis in INS-1 832/3-SNAP-GLP-1R cells labelled with SNAP-Surface 549, 100 nM agonist, 30-min incubation, size bars: 8 µm, representative images from *n*=2 experiments. (**F**) Comparison of ligand affinity with recycling rate using data from Figure 7A and Figure 7F, linear regression performed to determine goodness of fit. (**G**) Acute cAMP responses in INS-1 832/3 cells treated with each ligand and 500 µM IBMX for 10 minutes, normalised to exendin-asp3 response, *n*=4, 4-parameter fits of pooled data shown. (**H**) Alternative analysis of food intake data shown in Figure 7I, with intake over the first 30 min expressed relative to total intake over 8 hours, one-way ANOVA with Sidak's test. Data indicated as mean ± SEM or as individual replicates.
